# Memory recognition elicits autonomic-like responses in crayfish

**DOI:** 10.1242/jeb.249530

**Published:** 2025-06-18

**Authors:** Iván Oliver-Domínguez, Aidee Lashmi García-Kroepfly, Mireya Osorio-Palacios, Karina Mendoza-Ángeles, Jesús Hernández-Falcón

**Affiliations:** Laboratorio de Redes Neuronales, Departamento de Fisiología, Facultad de Medicina, Universidad Nacional Autónoma de México, CP 04510 Ciudad de México, México

**Keywords:** *Procambarus clarkii*, Cardiorespiratory activity, Hierarchy status, Physiological adaptations, Homeostasis

## Abstract

Organisms achieve homeostasis by making compensatory adjustments in response to changes in their internal and external environments. Such adjustments can be observed, for example, in variations of heart and respiratory rates triggered by different disturbances. In invertebrates, evidence of the existence of an autonomic nervous system structure has not been found. Even so, these animals show physiological responses – changes in cardiorespiratory activity (autonomic-like responses) – that maintain internal stability. In crustaceans, studies have found changes in both behavioural response and heart rate during memory processes. In the crayfish *Procambarus clarkii*, recognition memory has been behaviourally described when triads of these invertebrates interact under laboratory conditions and establish a hierarchical order (a dominant animal and two submissives). The main purpose of this work was to characterize the cardiorespiratory autonomic-like responses of *P. clarkii* during a 5-day recognition memory protocol. Our findings indicate significant differences in cardiorespiratory activity between day 1 (start of the memory protocol) and day 5 (when recognition memory is consolidated). Notably, there are differences based on hierarchy status, suggesting that the physiological response to recognition differs between dominant and submissive animals. This indicates that the retrieval of long-term recognition memory may lead to changes in autonomic-like responses.

## Teaser

**Highlighted Article:** The consolidation of a recognition memory in crayfish leads to adaptations in autonomic-like responses, such as heart and respiratory rates.

## INTRODUCTION

Compensatory adjustments of organisms in response to internal and external stimuli are necessary for the maintenance of homeostasis (Horn and Swanson, 2013). These adjustments occur, for example, in heart and respiratory rates in response to different disturbances (Shimizu and Okabe, 2007). In vertebrates, the sympathetic and parasympathetic divisions of the autonomic nervous system (ANS) regulate various structures, among others the heart muscle, resulting in an appropriate internal environment that allows the individual to react to possible external threats (McCorry, 2007). Cardiovascular system activity, particularly fluctuations in heart rate, is one of the key adjustments linked to attentional and memory processes in mammals, particularly those related to stress and fear (Hansen et al., 2003; Larra et al., 2014; Philippens et al., 2021).

In invertebrates, whose nervous systems range from simple ganglia networks to systems with central and peripheral components (Hartenstein, 2016), there is still no evidence of an ANS structure (Arbas et al., 2011). Nevertheless, these animals exhibit physiological responses that maintain internal stability (Shuranova et al., 2006; Hernández-Falcón et al., 2005). Crustaceans, for example, display ‘fight or flight’ behaviour during social interactions (Canero and Hermitte, 2014), accompanied by changes in cardiorespiratory activity (autonomic-like responses) (Schapker et al., 2002), typically attributed to the sympathetic division of the ANS in vertebrates. Furthermore, in crabs, according to Wilkens et al. (1974), the changes in cardiac activity are almost always accompanied by changes in respiratory activity. In other words, these responses in cardiorespiratory activity presented by Crustacea are similar to those presented by vertebrates owing to the ANS.

Several studies have investigated concurrent behavioural and cardiovascular changes in crustaceans during classical and fear conditioning (Burnovicz and Hermitte, 2010; Hermitte and Maldonado, 2006). These studies found that memory processes in crabs result in changes in both behavioural responses and heart rate. From an evolutionary perspective, studying these processes in invertebrates, such as crayfish, provides valuable insights into the origins and development of regulatory mechanisms that maintain homeostasis. Invertebrates, with their simpler nervous systems, offer a unique opportunity to explore fundamental physiological processes that are conserved across species, including vertebrates.

The crayfish *Procambarus clarkii* is a widely used decapod crustacean model for studying social interactions (Delgado-Morales et al., 2004). A 5-day protocol has been employed to behaviourally describe recognition memory in this invertebrate, where triads of crayfish interact under laboratory conditions to establish a hierarchical order, one dominant and two submissive individuals (submissive 1 and 2, respectively), with the submissive 1 showing a ‘double behaviour’, submissive to the dominant but dominant to the submissive 2 (Jiménez-Morales et al., 2018). Furthermore, the ability to simultaneously record cardiac and respiratory electrical activities in crayfish without affecting their function or behaviour (Osorio-Palacios et al., 2021) makes it an excellent model for studying autonomic-like responses in cardiorespiratory activities under different conditions (Schapker et al., 2002; Osorio-Palacios et al., 2021; Arellano-Tirado, 2016).

The main purpose of this work was to characterize the autonomic-like cardiorespiratory responses of triads of the crayfish *P. clarkii* during a 5-day recognition memory protocol, using electrophysiological recordings of each individual within the triad.

## MATERIALS AND METHODS

### Animals

We used triads of unrestrained adult male crayfish *Procambarus clarkii* (Girard 1852) in intermolt state (*n*=6), weighing 25–30 g (dry mass), obtained from a local provider. Upon arrival at the laboratory, animals were housed individually in aquaria (30×15×10 cm) with 5 cm of tap water, maintained at ambient temperature (20°C), under 12 h:12 h light:dark cycles (lights on at 07:00 h and lights off at 19:00 h). Animals were fed twice a week with cat chow (Whiskas).

### Behavioural and electrophysiological recordings

#### Cardiac electrical activity

To record cardiac electrical activity, we implanted electrodes in the pericardial sinus following a previously described procedure (Osorio-Palacios et al., 2021). Briefly, a hole was made in the pericardial sinus of cold-anesthetized crayfish. A PVC tube (Intramedic, 7426, Sweden) carrying a Pt-Ir Teflon coated wire (A-M Systems, 787000, Sequim, WA, USA) with a diameter of 75 μm and a length of 2 mm was inserted through this hole and fixed to the exoskeleton with dental cement (Acrimin Autocurable, Mexico). This electrode served as the active electrode. Another electrode was introduced 5 mm away from the active one (rostrum to tail) and was used as a reference electrode.

#### Respiratory electrical activity

To record the respiratory electrical activity, we implanted electrodes in both gill chambers (Osorio-Palacios et al., 2021). We placed a PT-Ir Teflon coated (A-M Systems, 787000) hook electrode (75 µm diameter) into each gill chamber of cold-anesthetized crayfish (active electrodes) and introduced a third electrode through the lateral region of the exoskeleton (reference electrode). We fixed the electrode wires to the carapace with dental cement (Acrimin Autocurable).

#### Recognition memory protocol

**Fig. 1.**
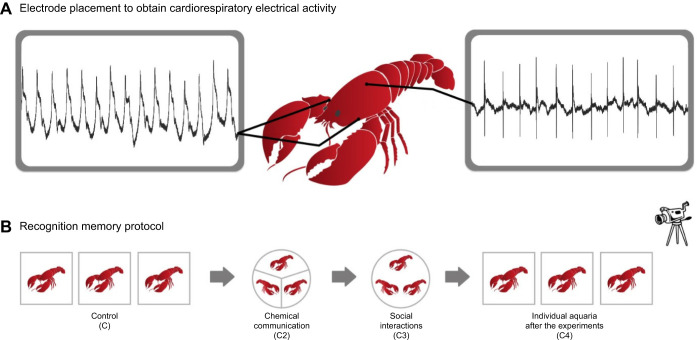
**Biological preparation.** (A) Schematic of an implanted crayfish. (B) Conditions in the 5-day protocol of recognition memory for each of the six triads. During the protocol, we videorecorded the triads’ behaviour and, simultaneously, recorded cardiorespiratory electrical activities.

Once implanted in the pericardial sinus and both gill chambers (Fig. 1A), we returned the crayfish to their individual aquaria and left them undisturbed for 48 h. Then we videorecorded the behaviour and simultaneously recorded the cardiorespiratory electrical activities from each triad following the 5-day memory protocol previously described in Jiménez-Morales et al. (2018). The protocol (Fig. 1B) was composed of the following conditions.

Control (C): each crayfish remained in their individual aquarium for 15 min.

Chemical communication (C2): the triad was placed in a circular plastic aquarium (40 cm diameter) filled with well-aerated tap water, 10 cm deep. Animals were initially separated by an opaque, Y-shaped plastic divider, which only allowed chemical communication, for 15 min.

Social interactions (C3): we removed the plastic divider, and the crayfish could interact freely for 45 min. Social interactions consisted of threats, attacks, fights (positive contacts), retreats and avoidance (negative contacts) performed by each crayfish (Jiménez-Morales et al., 2018). In a given triad, an animal threatens, attacks and fights with the other two until the opponent retreats or escapes. As time goes on, a dominant animal appears. During the first day of interactions, contacts are numerous and then they decline in subsequent days until they reach their minimum (Delgado-Morales et al., 2004).

Individual aquaria after the experiments (C4): we returned the animals to their individual aquaria where a recording was performed for 15 more minutes. We kept the crayfish in their individual aquaria until the next day.

We repeated this procedure for five consecutive days. The experiments were performed between 13:00 and 14:30 h.

### Signal acquisition

We amplified the electrical signals with AC amplifiers (A-M Systems) and in parallel sampled at 1000 Hz with an A/D converter (National Instruments, NI-USB-6211, Austin, TX, USA). We band-pass filtered the electrical signals between 0.3 and 100 Hz. We acquired all data using a MATLAB software (MathWorks, Natick, MA, USA) based algorithm developed in our laboratory and stored on a personal computer for off-line analysis. The experiments were approved by the Ethical Committee of the Faculty of Medicine at UNAM (FMED/DI/023/2018).

### Data analysis

#### Behavioural analysis

We categorized the dominant or submissive 1 or 2 status according to the method described elsewhere (Bovbjerg, 1953). Briefly, to analyze this behaviour, an independent observer studied each video, counting each positive and negative contact performed by a given animal, and noting down which of the other two received it. Each action was assigned a unit value and a unique code. The order of dominance is determined by the relationship between the number of positive and negative contacts, the dominant crayfish is the one that has a higher ratio of positive/negative contacts.

#### Electrophysiological analysis

We determined the cardiac and respiratory frequencies in 1-min moving windows for each member of the triads in all conditions (C, C2, C3 and C4) during the 5-day recognition memory protocol. Then we calculated the mean of each frequency for all conditions and every hierarchy (dominant, submissive 1 and submissive 2) during the 5 days of the recognition memory protocol, using MATLAB software.

#### Statistical analysis

To determine whether there were differences in heart and respiratory rates during the recognition memory protocol between hierarchies and days in every condition, we used Kruskal–Wallis (comparison between hierarchies every day) and Friedman tests (comparison between the hierarchies along the 5 days) with Dunn’s *post hoc* test (Tables S1–S4). We applied the Holm–Bonferroni correction for multiple comparisons. Differences were considered significant when *P*<0.05. We used R software to analyze the data [using the packages dplyr (https://CRAN.R-project.org/package=dplyr), tidyr (https://CRAN.R-project.org/package=tidyr), rstatix (https://CRAN.R-project.org/package=rstatix) and FSA (https://CRAN.R-project.org/package=FSA)] and construct the graphs (Wickham, 2016).

## RESULTS

### Cardiac activity

**Fig. 2.**
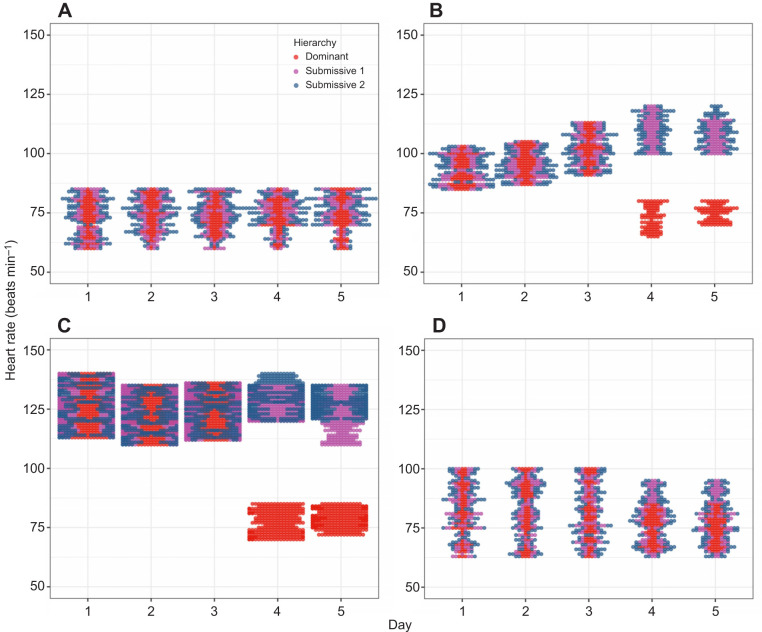
**Heart rate (*n*=6) for dominant (red dots), submissive 1 (purple dots) and submissive 2 (blue dots) crayfish.** Data are shown for the (A) control condition (C), (B) chemical communication (C2), (C) social interaction (C3) and (D) individual aquaria after the experiments (C4) across the 5-day recognition memory protocol.

To analyze the general behaviour of the heart rate (HR) in beats min^−1^, we constructed beeswarm plots for all crayfish, allowing us to visualize individual data points across all experimental conditions (Fig. 2). For C, the data points for the different hierarchies (dominant, submissive 1, and submissive 2) were intermixed throughout the 5-day period, indicating no apparent changes in HR between hierarchies or across days (Fig. 2A). For C2, during the first 3 days, the data points were similarly mixed. However, on days 4 and 5, the HR of dominant crayfish diverged from that of submissive individuals (Fig. 2B). The same pattern of C2 was also observed during the social interactions condition (Fig. 2C). For C4, when crayfish were returned to their individual aquaria, the HR data points were again intermixed across all hierarchies and days (Fig. 2D).

**Fig. 3.**
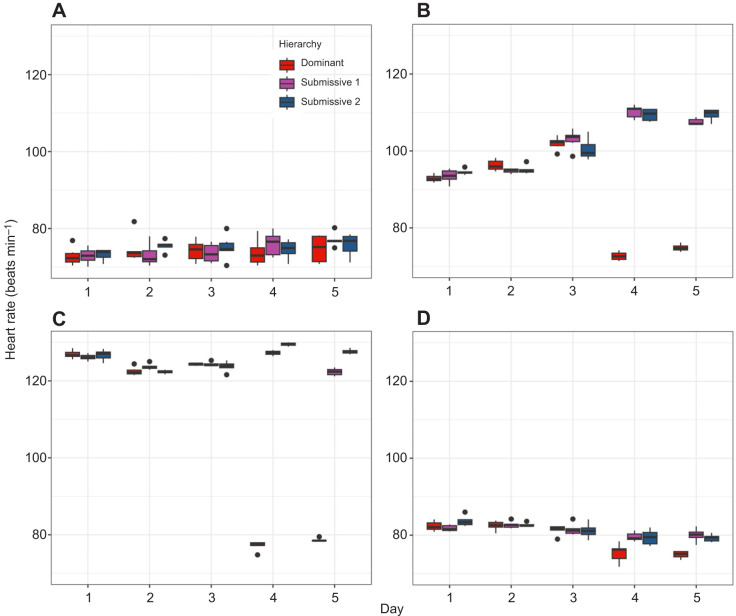
**Boxplots of the mean heart rate (*n*=6) for dominant (red dots), submissive 1 (purple dots) and submissive 2 (blue dots) crayfish.** Data are shown for the (A) control condition (C), (B) chemical communication (C2), (C) social interaction (C3) and (D) individual aquaria after the experiments (C4) across the 5-day recognition memory protocol.

**Fig. 4.**
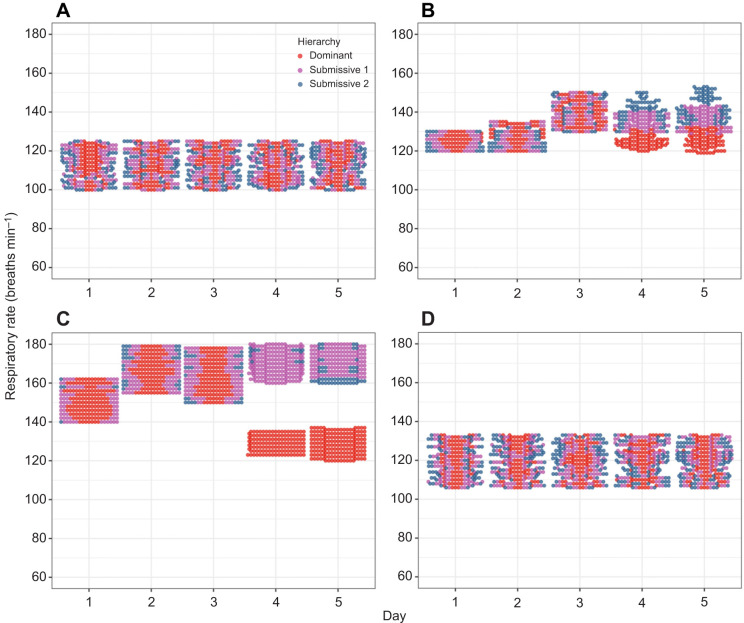
**Respiratory rate (*n*=6) for gill chamber 1 of dominant (red dots), submissive 1 (purple dots)and submissive 2 (blue dots) crayfish.** Data are shown for the (A) control condition (C), (B) chemical communication (C2), (C) social interaction (C3) and (D) individual aquaria after the experiments (C4) across the 5-day recognition memory protocol.

To further analyze these trends, we constructed boxplots of the mean HR to compare hierarchies within each day and across the 5-day period for each condition (Fig. 3). For C, no significant differences in HR were observed either between days (comparing each crayfish over time) or between hierarchies (comparing crayfish of the same hierarchy across days). This suggests that crayfish in their individual aquaria maintain a relatively stable HR, with a mean of 73±7.5 beats min^−1^ (Fig. 3A). For C2, when comparing hierarchies within the same day, significant differences in HR (*P*<0.05) were observed only on days 4 and 5 between dominant and submissive crayfish. Comparing the same hierarchies across days, significant differences were found in dominant crayfish between the first 3 days and the last 2 days, with HR decreasing in the latter period. In contrast, submissive crayfish showed increased HR on days 4 and 5 compared with days 1 and 2 (Fig. 3B). For C3, significant differences in HR (*P*<0.05) were observed between hierarchies on days 4 and 5, with dominant crayfish showing lower HR compared with both submissive individuals. Additionally, submissive 1 and 2 exhibited differences in HR on these days. Across the 5-day period, dominant crayfish showed a decrease in HR on days 4 and 5 compared with the first 3 days, wherease submissive crayfish maintained elevated HR levels, with some variations in the later days (Fig. 3C). Finally, in C4, significant differences (*P*<0.05) were observed only on days 4 and 5 between dominant and submissive crayfish. Dominant crayfish showed lower HR on days 4 and 5 compared with the first 3 days, whereas submissive 1 and 2 exhibited increased HR in the latter period (Fig. 4D).

For a comprehensive overview of all comparisons between days and hierarchies across conditions, refer to Table S1.

### Respiratory activity

**Fig. 5.**
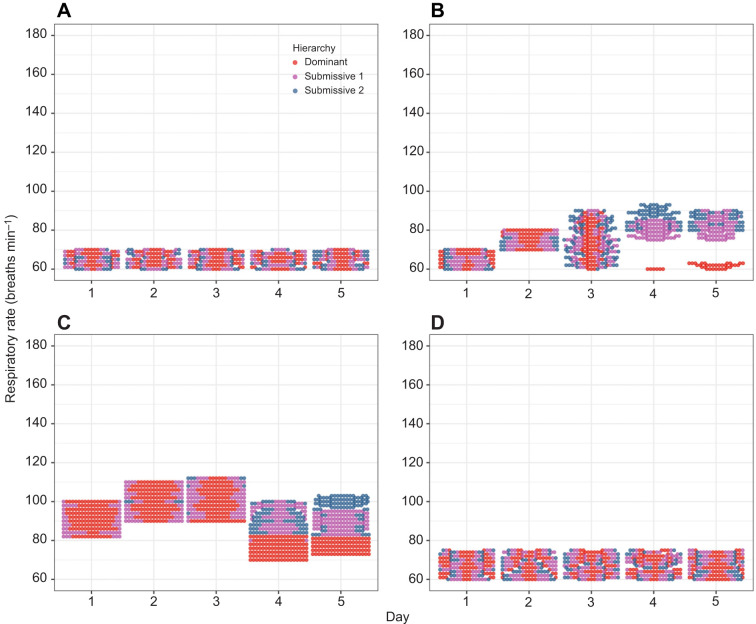
**Respiratory rate (*n*=6) for gill chamber 2 of dominant (red dots), submissive 1 (purple dots) and submissive 2 (blue dots) crayfish.** Data are shown for the (A) control condition (C), (B) chemical communication (C2), (C) social interaction (C3) and (D) individual aquaria after the experiments (C4) across the 5-day recognition memory protocol.

To analyze the general behaviour of respiratory rate (RR) in breaths min^−1^, we constructed beeswarm plots for both gill chambers (Figs 4 and 5) across all crayfish, allowing us to visualize individual data points in all experimental conditions. In C, the data points for the different hierarchies (dominant, submissive 1 and submissive 2) were intermixed throughout the 5-day period in both gill chambers (Figs 4A and 5A), indicating no apparent changes in RR between hierarchies or across days. In C2, during the first 3 days, the data points were similarly mixed in both gill chambers. However, on days 4 and 5, the RR of dominant crayfish diverged from that of submissive individuals in gill chamber 2 (Fig. 5B). In gill chamber 1 (Fig. 4B), this divergence was less pronounced, but a distinct grouping of dominant crayfish was noted. In C3, during the first 3 days, the data points were mixed in both gill chambers. On days 4 and 5, the RR of dominant crayfish diverged from that of submissive individuals in gill chamber 1 (Fig. 4C), whereas the points remained mixed in gill chamber 2 (Fig. 5C). And in C4, when crayfish were returned to their individual aquaria, the RR data points were again intermixed across all hierarchies and days in both gill chambers (Figs 4D and 5D).

**Fig. 6.**
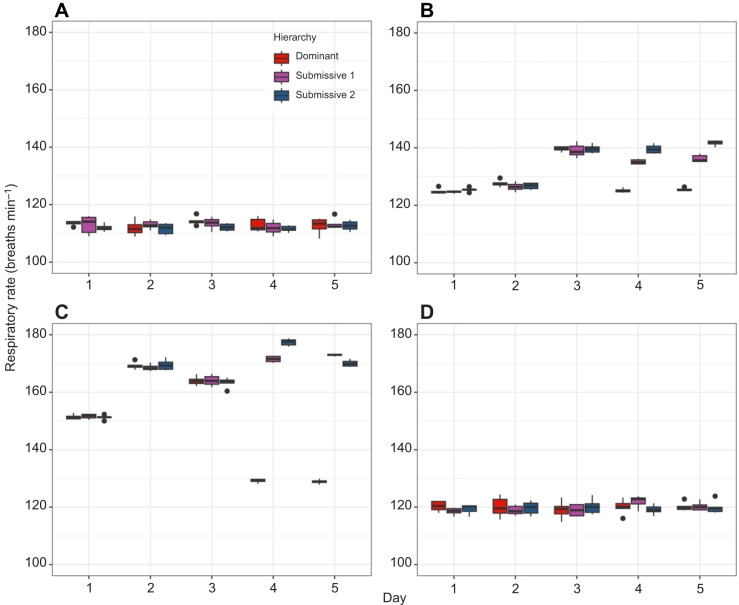
**Boxplots of respiratory rate (*n*=6) for gill chamber 1 of dominant (red), submissive 1 (purple) and submissive 2 (blue) crayfish.** Data are shown for the (A) control condition (C), (B) chemical communication (C2), (C) social interaction (C3) and (D) individual aquaria after the experiments (C4) across the 5-day recognition memory protocol.

**Fig. 7.**
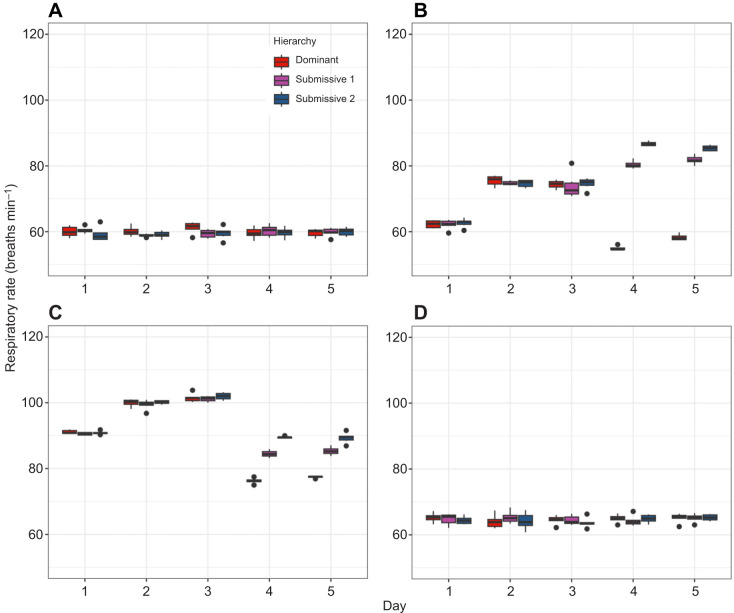
**Boxplots of respiratory rate (*n*=6) for gill chamber 2 of dominant (red), submissive 1 (purple) and submissive 2 (blue) crayfish.** Data are shown for the (A) control condition (C), (B) chemical communication (C2), (C) social interaction (C3) and (D) individual aquaria after the experiments (C4) across the 5-day recognition memory protocol.

To further analyze these trends, we constructed boxplots of the mean RR to compare hierarchies within each day and across the 5-day period for each condition (Figs 5 and 6). In C, no significant differences in RR were observed either between days (comparing each crayfish over time) or between hierarchies (comparing crayfish of the same hierarchy across days). This suggests that crayfish in their individual aquaria maintain a relatively stable RR, with a mean of 113±8.1 breaths min^−1^ in gill chamber 1 (Fig. 6A) and 73±7.8 breaths min^−1^ in gill chamber 2 (Fig. 7A). In C2, when comparing hierarchies within the same day, significant differences in RR (*P*<0.05) were observed only on days 4 and 5 between dominant and submissive crayfish, as well as between submissives 1 and 2, in both gill chambers (Figs 6B and 7B). Comparing the same hierarchies across days, significant differences were found in dominant crayfish between the first 2 days and day 3, and between day 3 and days 4–5 in gill chamber 1. In gill chamber 2, significant differences were observed between the first 3 days compared with days 4–5. Submissives 1 and 2 showed increased RR on days 4 and 5 compared with earlier days in gill chamber 2, whereas in gill chamber 1, the RR of dominant crayfish decreased to levels similar to the first 2 days, and submissive crayfish maintained elevated RR levels. In C3, significant differences in RR (*P*<0.05) were observed between hierarchies on days 4 and 5, with dominant crayfish showing lower RR compared with both submissive individuals in both gill chambers. Across the 5-day period, dominant crayfish showed a decrease in RR on days 4 and 5 compared with the first 3 days, whereas submissive crayfish exhibited increased RR in the latter period in gill chamber 1 (Fig. 6C). In gill chamber 2 (Fig. 7C), submissive crayfish showed a decreased RR on days 4 and 5 compared with days 2 and 3. Finally, in C4, no significant differences in RR were observed between days or hierarchies when crayfish were returned to their individual aquaria (Figs 6D and 7D).

For a comprehensive overview of all comparisons between days and hierarchies across conditions, refer to Table S2.

### Cardiorespiratory activity

**Fig. 8.**
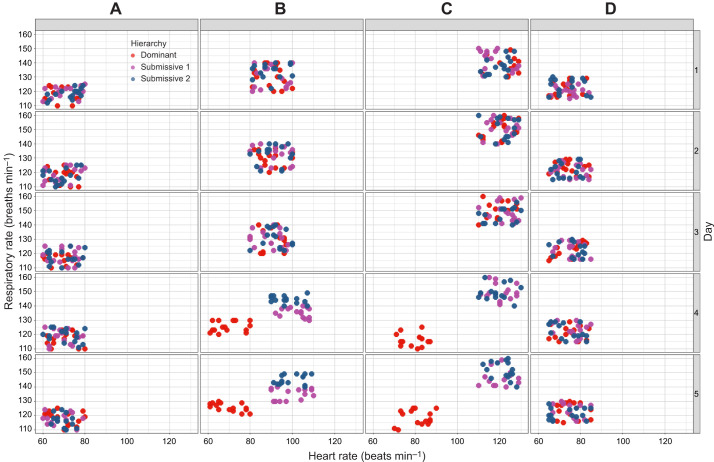
**Scatter plot of heart rate (*x*-axis) and respiratory rate (*y*-axis) for one triad across all conditions (columns) during the 5-day (rows) recognition memory protocol.** Dominant crayfish are represented by red dots, submissive 1 by purple dots and submissive 2 by blue dots. RR1, respiratory rate from gill chamber 1.

To visualize the autonomic-like responses in HR and RR throughout the memory protocol, we simultaneously evaluated both activities and constructed a scatter plot of the mean HR and RR (from gill chamber 1, where values are closest to HR) for one example triad across all conditions (Fig. 8).

For both C and C4, HR and RR values remained consistent over the 5-day period, with no significant differences between hierarchies. For C2 and C3, during the first 3 days, HR and RR values were similar between dominant and submissive crayfish within each condition. However, on days 4 and 5, the HR and RR of dominant crayfish diverged from those of submissive individuals, indicating hierarchical differentiation in autonomic-like responses.

### Comparison of day 1 and day 5

**Fig. 9.**
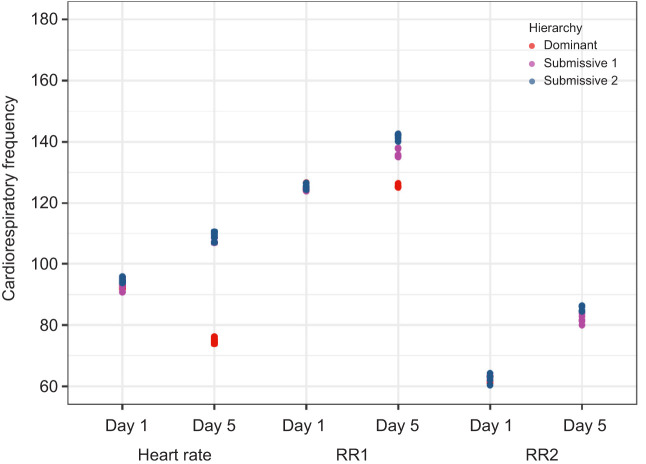
**Scatter plot of the mean heart rate and respiratory rate (RR1 and RR2) (*n*=6) during day 1 and day 5 of the memory protocol under chemical communication (C2).** RR2, respiratory rate from gill chamber 2.

**Fig. 10.**
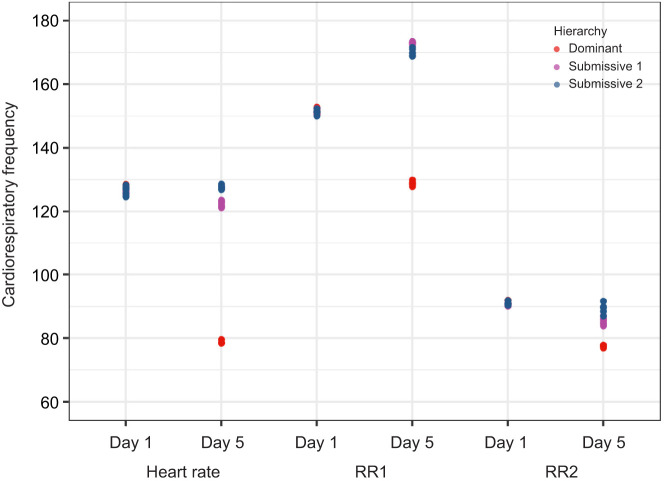
Scatter plot of the mean heart rate and respiratory rate (RR1 and RR2) (*n*=6) during day 1 and day 5 of the memory protocol under social interactions (C3).

To further analyze the changes in cardiorespiratory activity in the memory protocol, we compared the mean HR and RR for all triads (*n*=6) on day 1 (start of the memory protocol) and day 5 (when recognition memory is consolidated) during the chemical communication (C2) (Fig. 9) and social interaction (C3) (Fig. 10) conditions.

For C2, on day 1, HR values were homogeneous across all crayfish, ranging between 80 and 100 breaths min^−1^. RR values in both gill chambers showed distinct frequencies (one above 120 breaths min^−1^ and the other below 80 breaths min^−1^), but no separation between dominant and submissive crayfish was observed. On day 5, HR values split into two groups: dominant crayfish exhibited lower HR (below 80 breaths min^−1^), whereas submissive crayfish showed higher HR (100–120 breaths min^−1^). RR values also showed a broader distribution, with dominant crayfish displaying distinct HR and RR values compared with submissive individuals. Hierarchical groupings based on RR values became evident.

For C3, on day 1, HR (110–130 beats min^−1^) and RR (140–160 breaths min^−1^ in one chamber and 80–100 breaths min^−1^ in the other) were similar across all crayfish, regardless of social status. On day 5, the HR of submissive crayfish remained similar to day 1 values, whereas dominant crayfish showed lower HR (below 80 beats min^−1^). RR increased to over 160 breaths min^−1^ in submissive crayfish in one chamber, with clear separation between dominant and submissive individuals. The second chamber showed no significant changes.

## DISCUSSION

Our results align with previous research on autonomic-like responses during agonistic encounters in crayfish (Schapker et al., 2002). Although earlier studies demonstrated increases in HR and RR in response to conspecific detection, our study extends this understanding by employing a 5-day memory protocol to explore how these autonomic-like responses evolve as recognition memory consolidates. Unlike previous work, which focused primarily on immediate physiological changes during social interactions, we investigated the long-term changes in cardiorespiratory activity as crayfish establish and maintain hierarchical relationships over time.

### Key findings and their implications

Our findings reveal significant differences in cardiorespiratory activity between day 1 (the start of the memory protocol) and day 5 (when recognition memory is consolidated, as described by Jiménez-Morales et al., 2018). This suggests that the consolidation of recognition memory drives changes in autonomic-like responses. Notably, these changes vary depending on hierarchical status, with dominant and submissive crayfish exhibiting distinct physiological profiles. For example, dominant crayfish showed lower HR and RR on days 4 and 5, whereas submissive individuals displayed elevated rates, indicating a heightened sympathetic-like response to the presence of dominants.

Interestingly, the ‘double behaviour’ of submissive 1 crayfish – submissive to the dominant but dominant over submissive 2 – was also reflected in their cardiorespiratory activity. Their HR and RR values were intermediate between those of dominant and submissive 2 crayfish, mirroring their behavioural role (Delgado-Morales et al., 2004; Jiménez-Morales et al., 2018). This finding enhances the intricate link between social behaviour and physiological regulation in crayfish.

### Sensory mechanisms and hierarchical establishment

The changes in HR and RR observed on days 4 and 5 in both experimental conditions (C2 and C3) highlight the critical role of olfactory sensory inputs in crayfish. As previously reported, chemical communication plays a central role in modifying cardiorespiratory activity and establishing social hierarchies (Delgado-Morales et al., 2004). Our results suggest that the detection of dominant individuals through chemical cues triggers a sustained autonomic-like response in submissive crayfish, even in the absence of direct physical interactions. This aligns with the idea that sensory information, particularly olfactory inputs, reaches brain centres responsible for autonomic control, eliciting a homeostatic response tailored to the social context (Ashby and Larimer, 1965).

### Resilience in dominant crayfish

The data also point to a greater resilience in dominant crayfish, who maintain stable autonomic values once recognition memory is consolidated. This stability may reflect their established social position, reducing the need for continuous physiological adjustments. In contrast, submissive crayfish exhibit heightened HR and RR, likely because of the ongoing stress of navigating their subordinate role. This divergence in physiological responses emphasizes the adaptive nature of autonomic regulation in social species.

### Comparative perspectives

The changes in HR and RR observed in crayfish during social interactions are not solely attributable to physical contact (Schapker et al., 2002). Even in the absence of direct interactions, the mere presence of a dominant individual was sufficient to trigger autonomic-like responses in submissive crayfish. This phenomenon mirrors findings in vertebrates, where social stress and recognition memory similarly influence autonomic activity. For instance, studies in rats (Richardson and Campbell, 1991; Miasnikov et al., 2006), mice (Liu et al., 2013; Stiedl and Spiess, 1997) and marmoset monkeys (Philippens et al., 2021) have demonstrated that memory consolidation is accompanied by changes in HR and RR, suggesting a conserved mechanism across species.

### Differentiated autonomic responses

Another intriguing observation is the differential RR patterns between the two gill chambers. Although both chambers showed similar trends, one consistently exhibited higher RR than the other. This asymmetry may indicate specialized functions or differential autonomic control of each gill chamber, a phenomenon also observed during sleep in crayfish (Osorio-Palacios et al., 2021; Mendoza-Angeles et al., 2025). Further research could explore whether this asymmetry is linked to specific environmental or social cues.

### Stress, memory and evolutionary implications

The stress experienced by crayfish during social interactions may play a crucial role in enhancing learning and memory formation. As noted by Rivi et al. (2023), long-term memory formation requires significant neuronal resources, and organisms are likely to invest energy only in events deemed biologically relevant. The stress associated with social hierarchy establishment may serve as a signal of relevance, promoting memory consolidation. Additionally, the autonomic-like responses observed during chemical communication (C2) on days 4 and 5 may be linked to anxiety-like behaviours, as described in Fossat et al. (2014). Investigating the emotional and stress-related dimensions of these responses could provide valuable insights into the evolutionary origins of emotions in invertebrates, a field that remains in its early stages (Rivi et al., 2023; Perry and Baciadonna, 2017).

From an evolutionary perspective, it would be fascinating to explore the brain regions involved in regulating these autonomic-like responses. For example, the circumesophageal connectives in crustaceans, which modulate both scaphognathite (respiratory) and heart rhythms simultaneously in about 70% of cases (Wilkens et al., 1974), could be a key area of study. Understanding how these neural pathways integrate sensory and social information to regulate cardiorespiratory activity could shed light on the evolution of autonomic control systems in both invertebrates and vertebrates.

### Conclusions

Our study demonstrates that crayfish exhibit autonomic-like responses that adapt over a 5-day memory protocol, with recognition memory influencing physiological variables such as HR and RR. These responses vary according to hierarchical status, with submissive crayfish showing greater increases in HR and RR in the presence of dominants, even without physical contact. In other words, the consolidation of recognition memory is associated with changes in cardiorespiratory activity and points to the existence of a sympathetic-like and parasympathetic-like regulatory system in these invertebrates. Future research could explore the neural mechanisms underlying these responses and their evolutionary significance, offering new insights into the origins of autonomic regulation and social behaviour.

## Supplementary Material

10.1242/jexbio.249530_sup1 Supplementary information
