# Supplementary material for: Memory recognition elicits autonomic-like responses in crayfish
Source: J Exp Biol. 2025 Jun 18;228(12):jeb249530. doi: 10.1242/jeb.249530 (PMC12211588; doi:10.1242/jeb.249530)
Supplement: Supplementary information [file jexbio-228-249530-s1.pdf]

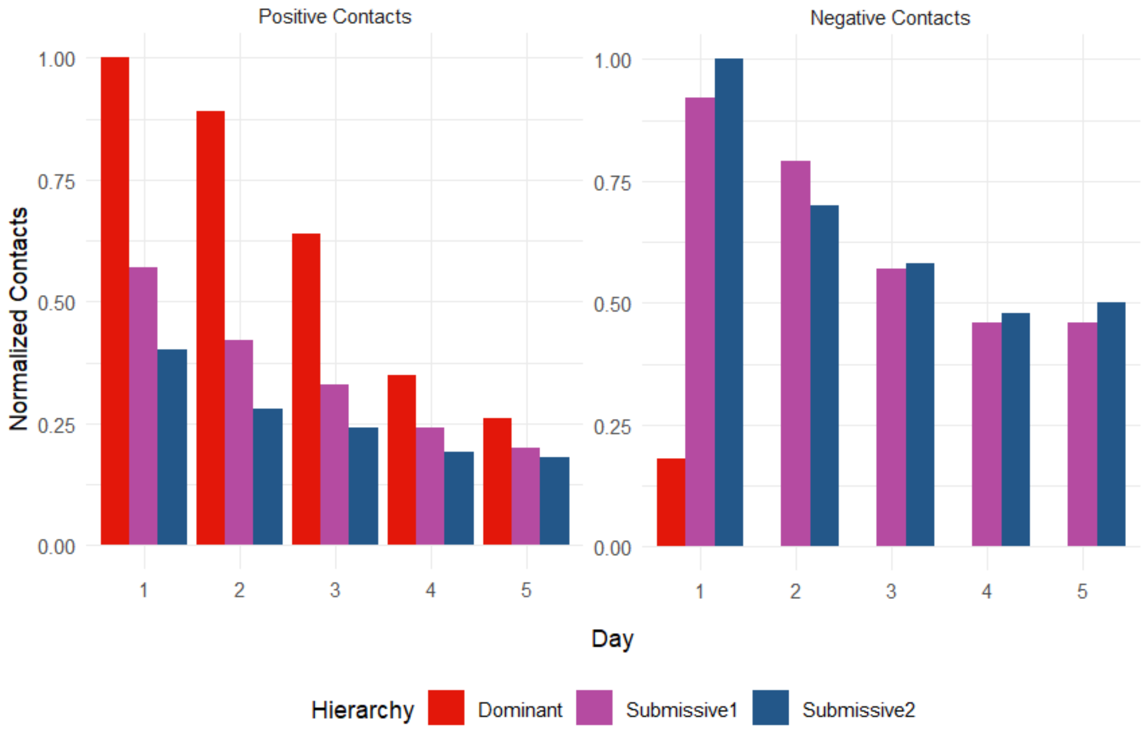

Fig. S1. Normalized negative (retreats and avoidances) and positive (threats, attacks and fights) contacts (n = 6) for dominant (red bars), submissive 1 (purple bars), and submissive 2 (blue bars) crayfish. Data are shown for the social interactions condition (C3) in the 5-day protocol of memory recognition.

Table S1. p-values of the comparisons in the increments in heart rate.

Experimental day	Subject	Experimental day	Subject	p-value
C2				
1	Submissive 1	1	Dominant	0.98886
1	Submissive 2	1	Dominant	0.9999995
1	Submissive 1	2	Dominant	0.9980436
1	Submissive 2	2	Dominant	1
1	Submissive 1	3	Dominant	0.0024843
1	Submissive 2	3	Dominant	0.0315261
1	Submissive 1	4	Dominant	0.0002648
1	Submissive 2	4	Dominant	0.0000118
1	Submissive 1	5	Dominant	0.004536
1	Submissive 2	5	Dominant	0.0002648
1	Submissive 2	1	Submissive 1	0.9999706
1	Submissive 2	2	Submissive 1	0.9384139
1	Submissive 2	3	Submissive 1	0.0924296
1	Submissive 2	4	Submissive 1	0.0000002
1	Submissive 2	5	Submissive 1	0.0123915
2	Dominant	1	Dominant	1
2	Submissive 1	1	Dominant	0.9987087
2	Submissive 2	1	Dominant	1
2	Submissive 1	2	Dominant	0.9918098
2	Submissive 2	2	Dominant	0.999997
2	Submissive 1	3	Dominant	0.7864741
2	Submissive 2	3	Dominant	0.3466523
2	Submissive 1	4	Dominant	0
2	Submissive 2	4	Dominant	0.0000002
2	Submissive 1	5	Dominant	0.0000003
2	Submissive 2	5	Dominant	0.0000048
2	Submissive 1	1	Submissive 1	0.4904964
2	Submissive 2	1	Submissive 1	0.8943568
2	Submissive 2	2	Submissive 1	0.999997
2	Submissive 2	3	Submissive 1	0.6141069
2	Submissive 2	4	Submissive 1	0.0000141
2	Submissive 2	5	Submissive 1	0.1900306
2	Submissive 2	1	Submissive 2	0.9994813
3	Dominant	1	Dominant	0.14145
3	Submissive 1	1	Dominant	0.3206688
3	Submissive 2	1	Dominant	0.83456
3	Dominant	2	Dominant	0.082658
3	Submissive 1	2	Dominant	0.208676

Journal of Experimental Biology • Supplementary information

3	Submissive 2	2	Dominant	0.7042921
3	Submissive 1	3	Dominant	1
3	Submissive 2	3	Dominant	0.9971079
3	Submissive 1	4	Dominant	0
3	Submissive 2	4	Dominant	0
3	Submissive 1	5	Dominant	0
3	Submissive 2	5	Dominant	0
3	Submissive 1	1	Submissive 1	0.0093619
3	Submissive 2	1	Submissive 1	0.0924296
3	Submissive 1	2	Submissive 1	0.9497185
3	Submissive 2	2	Submissive 1	0.9999435
3	Submissive 2	3	Submissive 1	0.9999706
3	Submissive 2	4	Submissive 1	0.0039092
3	Submissive 2	5	Submissive 1	0.974693
3	Submissive 2	1	Submissive 2	0.4602582
3	Submissive 2	2	Submissive 2	0.974693
4	Dominant	1	Dominant	0.0000011
4	Submissive 1	1	Dominant	0.0000023
4	Submissive 2	1	Dominant	0.0163069
4	Dominant	2	Dominant	0.0000028
4	Submissive 1	2	Dominant	0.0000009
4	Submissive 2	2	Dominant	0.0081203
4	Dominant	3	Dominant	0
4	Submissive 1	3	Dominant	0.127598
4	Submissive 2	3	Dominant	0.9999706
4	Submissive 1	4	Dominant	0
4	Submissive 2	4	Dominant	0
4	Submissive 1	5	Dominant	0
4	Submissive 2	5	Dominant	0
4	Submissive 1	1	Submissive 1	0
4	Submissive 2	1	Submissive 1	0.0001353
4	Submissive 1	2	Submissive 1	0.0001896
4	Submissive 2	2	Submissive 1	0.2721701
4	Submissive 1	3	Submissive 1	0.0458879
4	Submissive 2	3	Submissive 1	0.9971079
4	Submissive 2	4	Submissive 1	0.5521307
4	Submissive 2	5	Submissive 1	0.9999999
4	Submissive 2	1	Submissive 2	0.0024843
4	Submissive 2	2	Submissive 2	0.0584044
4	Submissive 2	3	Submissive 2	0.8112797
5	Dominant	1	Dominant	0.0000287
5	Submissive 1	1	Dominant	0.0657026
5	Submissive 2	1	Dominant	0.0000811

Journal of Experimental Biology • Supplementary information

5	Dominant	2	Dominant	0.0000683
5	Submissive 1	2	Dominant	0.0357912
5	Submissive 2	2	Dominant	0.0000341
5	Dominant	3	Dominant	0
5	Submissive 1	3	Dominant	1
5	Submissive 2	3	Dominant	0.6141069
5	Dominant	4	Dominant	0.9999706
5	Submissive 1	4	Dominant	0
5	Submissive 2	4	Dominant	0
5	Submissive 1	5	Dominant	0
5	Submissive 2	5	Dominant	0
5	Submissive 1	1	Submissive 1	0.0008303
5	Submissive 2	1	Submissive 1	0.0000003
5	Submissive 1	2	Submissive 1	0.5831713
5	Submissive 2	2	Submissive 1	0.004536
5	Submissive 1	3	Submissive 1	0.9999932
5	Submissive 2	3	Submissive 1	0.3466523
5	Submissive 1	4	Submissive 1	0.2497483
5	Submissive 2	4	Submissive 1	0.9998959
5	Submissive 2	5	Submissive 1	0.8112797
5	Submissive 2	1	Submissive 2	0.0000083
5	Submissive 2	2	Submissive 2	0.0004344
5	Submissive 2	3	Submissive 2	0.0584044
5	Submissive 2	4	Submissive 2	0.974693
C3				
1	Submissive 1	1	Dominant	1
1	Submissive 2	1	Dominant	0.9996449
1	Submissive 1	2	Dominant	1
1	Submissive 2	2	Dominant	0.9807189
1	Submissive 1	3	Dominant	1
1	Submissive 2	3	Dominant	0.9999994
1	Submissive 1	4	Dominant	0
1	Submissive 2	4	Dominant	0
1	Submissive 1	5	Dominant	0
1	Submissive 2	5	Dominant	0
1	Submissive 2	1	Submissive 1	0.9994656
1	Submissive 2	2	Submissive 1	0.8911797
1	Submissive 2	3	Submissive 1	1
1	Submissive 2	4	Submissive 1	0.999982
1	Submissive 2	5	Submissive 1	0.9999094
2	Dominant	1	Dominant	1
2	Submissive 1	1	Dominant	0.9999454
2	Submissive 2	1	Dominant	1

Journal of Experimental Biology • Supplementary information

2	Submissive 1	2	Dominant	1
2	Submissive 2	2	Dominant	0.9999681
2	Submissive 1	3	Dominant	0.9958161
2	Submissive 2	3	Dominant	1
2	Submissive 1	4	Dominant	0
2	Submissive 2	4	Dominant	0
2	Submissive 1	5	Dominant	0
2	Submissive 2	5	Dominant	0
2	Submissive 1	1	Submissive 1	0.9999681
2	Submissive 2	1	Submissive 1	1
2	Submissive 2	2	Submissive 1	0.9977451
2	Submissive 2	3	Submissive 1	1
2	Submissive 2	4	Submissive 1	0.9845968
2	Submissive 2	5	Submissive 1	0.9707283
2	Submissive 2	1	Submissive 2	0.9999974
3	Dominant	1	Dominant	1
3	Submissive 1	1	Dominant	0.9999901
3	Submissive 2	1	Dominant	0.9983837
3	Dominant	2	Dominant	0.9999094
3	Submissive 1	2	Dominant	0.9969048
3	Submissive 2	2	Dominant	0.9999974
3	Submissive 1	3	Dominant	1
3	Submissive 2	3	Dominant	0.9707283
3	Submissive 1	4	Dominant	0
3	Submissive 2	4	Dominant	0
3	Submissive 1	5	Dominant	0
3	Submissive 2	5	Dominant	0
3	Submissive 1	1	Submissive 1	0.999982
3	Submissive 2	1	Submissive 1	0.9988613
3	Submissive 1	2	Submissive 1	0.9644644
3	Submissive 2	2	Submissive 1	1
3	Submissive 2	3	Submissive 1	0.8760558
3	Submissive 2	4	Submissive 1	0.2482849
3	Submissive 2	5	Submissive 1	0.1984858
3	Submissive 2	1	Submissive 2	0.7392114
3	Submissive 2	2	Submissive 2	0.9807189
4	Dominant	1	Dominant	0
4	Submissive 1	1	Dominant	0.9178317
4	Submissive 2	1	Dominant	1
4	Dominant	2	Dominant	0
4	Submissive 1	2	Dominant	0.6676387
4	Submissive 2	2	Dominant	0.999982
4	Dominant	3	Dominant	0

4	Submissive 1	3	Dominant	0.990501
4	Submissive 2	3	Dominant	1
4	Submissive 1	4	Dominant	0
4	Submissive 2	4	Dominant	0
4	Submissive 1	5	Dominant	0
4	Submissive 2	5	Dominant	0
4	Submissive 1	1	Submissive 1	0.9051042
4	Submissive 2	1	Submissive 1	1
4	Submissive 1	2	Submissive 1	0.4167038
4	Submissive 2	2	Submissive 1	0.9983837
4	Submissive 1	3	Submissive 1	0.9994656
4	Submissive 2	3	Submissive 1	1
4	Submissive 2	4	Submissive 1	0.9807189
4	Submissive 2	5	Submissive 1	0.9644644
4	Submissive 2	1	Submissive 2	0.9999948
4	Submissive 2	2	Submissive 2	1
4	Submissive 2	3	Submissive 2	0.9845968
5	Dominant	1	Dominant	0
5	Submissive 1	1	Dominant	0.8760558
5	Submissive 2	1	Dominant	0.8760558
5	Dominant	2	Dominant	0
5	Submissive 1	2	Dominant	0.5920778
5	Submissive 2	2	Dominant	0.5920778
5	Dominant	3	Dominant	0
5	Submissive 1	3	Dominant	0.9807189
5	Submissive 2	3	Dominant	0.9807189
5	Dominant	4	Dominant	0.9994656
5	Submissive 1	4	Dominant	0
5	Submissive 2	4	Dominant	0
5	Submissive 1	5	Dominant	0
5	Submissive 2	5	Dominant	0
5	Submissive 1	1	Submissive 1	0.8597437
5	Submissive 2	1	Submissive 1	0.8597437
5	Submissive 1	2	Submissive 1	0.3480045
5	Submissive 2	2	Submissive 1	0.3480045
5	Submissive 1	3	Submissive 1	0.9983837
5	Submissive 2	3	Submissive 1	0.9983837
5	Submissive 1	4	Submissive 1	1
5	Submissive 2	4	Submissive 1	1
5	Submissive 2	5	Submissive 1	1
5	Submissive 2	1	Submissive 2	0.9999094
5	Submissive 2	2	Submissive 2	0.9707283
5	Submissive 2	3	Submissive 2	0.1984858

Journal of Experimental Biology • Supplementary information

5	Submissive 2	4	Submissive 2	0.9644644
C4				
2	Dominant	1	Dominant	0.9975219
3	Dominant	1	Dominant	0.9920098
4	Dominant	1	Dominant	0.999989
5	Dominant	1	Dominant	1
1	Submissive 1	1	Dominant	1
2	Submissive 1	1	Dominant	1
3	Submissive 1	1	Dominant	0.9939079
4	Submissive 1	1	Dominant	0.983291
5	Submissive 1	1	Dominant	0.999989
1	Submissive 2	1	Dominant	0.8333874
2	Submissive 2	1	Dominant	1
3	Submissive 2	1	Dominant	0.9999942
4	Submissive 2	1	Dominant	0.9999986
5	Submissive 2	1	Dominant	0.983291
3	Dominant	2	Dominant	1
4	Dominant	2	Dominant	1
5	Dominant	2	Dominant	0.9999391
1	Submissive 1	2	Dominant	0.9954187
2	Submissive 1	2	Dominant	0.9998371
3	Submissive 1	2	Dominant	1
4	Submissive 1	2	Dominant	1
5	Submissive 1	2	Dominant	1
1	Submissive 2	2	Dominant	0.9999798
2	Submissive 2	2	Dominant	0.9975219
3	Submissive 2	2	Dominant	0.868592
4	Submissive 2	2	Dominant	0.9999997
5	Submissive 2	2	Dominant	1
4	Dominant	3	Dominant	0.9999994
5	Dominant	3	Dominant	0.9996064
1	Submissive 1	3	Dominant	0.9867756
2	Submissive 1	3	Dominant	0.9991298
3	Submissive 1	3	Dominant	1
4	Submissive 1	3	Dominant	1
5	Submissive 1	3	Dominant	0.9999994
1	Submissive 2	3	Dominant	0.9999986
2	Submissive 2	3	Dominant	0.9920098
3	Submissive 2	3	Dominant	0.7937071
4	Submissive 2	3	Dominant	0.9999942
5	Submissive 2	3	Dominant	1
5	Dominant	4	Dominant	1
1	Submissive 1	4	Dominant	0.9999644

Journal of Experimental Biology • Supplementary information

2	Submissive 1	4	Dominant	1
3	Submissive 1	4	Dominant	0.9999997
4	Submissive 1	4	Dominant	0.9999942
5	Submissive 1	4	Dominant	1
1	Submissive 2	4	Dominant	0.9966046
2	Submissive 2	4	Dominant	0.999989
3	Submissive 2	4	Dominant	0.983291
4	Submissive 2	4	Dominant	1
5	Submissive 2	4	Dominant	0.9999942
1	Submissive 1	5	Dominant	1
2	Submissive 1	5	Dominant	1
3	Submissive 1	5	Dominant	0.9997437
4	Submissive 1	5	Dominant	0.9987442
5	Submissive 1	5	Dominant	1
1	Submissive 2	5	Dominant	0.954078
2	Submissive 2	5	Dominant	1
3	Submissive 2	5	Dominant	0.9994086
4	Submissive 2	5	Dominant	1
5	Submissive 2	5	Dominant	0.9987442
2	Submissive 1	1	Submissive 1	1
3	Submissive 1	1	Submissive 1	0.9896565
4	Submissive 1	1	Submissive 1	0.974196
5	Submissive 1	1	Submissive 1	0.9999644
1	Submissive 2	1	Submissive 1	0.7937071
2	Submissive 2	1	Submissive 1	1
3	Submissive 2	1	Submissive 1	0.9999986
4	Submissive 2	1	Submissive 1	0.9999942
5	Submissive 2	1	Submissive 1	0.974196
3	Submissive 1	2	Submissive 1	0.9994086
4	Submissive 1	2	Submissive 1	0.9975219
5	Submissive 1	2	Submissive 1	1
1	Submissive 2	2	Submissive 1	0.9355347
2	Submissive 2	2	Submissive 1	1
3	Submissive 2	2	Submissive 1	0.9997437
4	Submissive 2	2	Submissive 1	1
5	Submissive 2	2	Submissive 1	0.9975219
4	Submissive 1	3	Submissive 1	1
5	Submissive 1	3	Submissive 1	0.9999997
1	Submissive 2	3	Submissive 1	0.9999971
2	Submissive 2	3	Submissive 1	0.9939079
3	Submissive 2	3	Submissive 1	0.814079
4	Submissive 2	3	Submissive 1	0.9999971
5	Submissive 2	3	Submissive 1	1

Journal of Experimental Biology • Supplementary information

5	Submissive 1	4	Submissive 1	0.9999942
1	Submissive 2	4	Submissive 1	0.9999999
2	Submissive 2	4	Submissive 1	0.983291
3	Submissive 2	4	Submissive 1	0.7269723
4	Submissive 2	4	Submissive 1	0.9999644
5	Submissive 2	4	Submissive 1	1
1	Submissive 2	5	Submissive 1	0.9966046
2	Submissive 2	5	Submissive 1	0.999989
3	Submissive 2	5	Submissive 1	0.983291
4	Submissive 2	5	Submissive 1	1
5	Submissive 2	5	Submissive 1	0.9999942
2	Submissive 2	1	Submissive 2	0.8333874
3	Submissive 2	1	Submissive 2	0.3780033
4	Submissive 2	1	Submissive 2	0.9920098
5	Submissive 2	1	Submissive 2	0.9999999
3	Submissive 2	2	Submissive 2	0.9999942
4	Submissive 2	2	Submissive 2	0.9999986
5	Submissive 2	2	Submissive 2	0.983291
4	Submissive 2	3	Submissive 2	0.9920098
5	Submissive 2	3	Submissive 2	0.7269723
5	Submissive 2	4	Submissive 2	0.9999644

Table S2. p-values of the comparisons in the increments in respiratory rate.

Experimental day	Subject	Experimental day	Subject	p-value
C2 chamber 1				
1	Submissive 1	1	Dominant	0.9997526
1	Submissive 2	1	Dominant	0.8732557
1	Submissive 1	2	Dominant	1
1	Submissive 2	2	Dominant	0.9891533
1	Submissive 1	3	Dominant	0.0838572
1	Submissive 2	3	Dominant	0.0056738
1	Submissive 1	4	Dominant	1
1	Submissive 2	4	Dominant	0.9993666
1	Submissive 1	5	Dominant	0.9990263
1	Submissive 2	5	Dominant	0.8122109
1	Submissive 2	1	Submissive 1	0.9998521
1	Submissive 2	2	Submissive 1	0.906399
1	Submissive 2	3	Submissive 1	0.0000853
1	Submissive 2	4	Submissive 1	0.0341754
1	Submissive 2	5	Submissive 1	0.1259402
2	Dominant	1	Dominant	0.9999999

2	Submissive 1	1	Dominant	1
2	Submissive 2	1	Dominant	0.9999936
2	Submissive 1	2	Dominant	1
2	Submissive 2	2	Dominant	0.9969232
2	Submissive 1	3	Dominant	0.4843604
2	Submissive 2	3	Dominant	0.9333776
2	Submissive 1	4	Dominant	0.9999868
2	Submissive 2	4	Dominant	0.9702671
2	Submissive 1	5	Dominant	1
2	Submissive 2	5	Dominant	0.9999995
2	Submissive 1	1	Submissive 1	0.9999145
2	Submissive 2	1	Submissive 1	0.9446316
2	Submissive 2	2	Submissive 1	0.9999744
2	Submissive 2	3	Submissive 1	0.2578589
2	Submissive 2	4	Submissive 1	0.9985391
2	Submissive 2	5	Submissive 1	0.9999987
2	Submissive 2	1	Submissive 2	0.4284278
3	Dominant	1	Dominant	0.5419537
3	Submissive 1	1	Dominant	0.0484455
3	Submissive 2	1	Dominant	0.6289186
3	Dominant	2	Dominant	0.2375598
3	Submissive 1	2	Dominant	0.0110909
3	Submissive 2	2	Dominant	0.3017179
3	Submissive 1	3	Dominant	0.9978573
3	Submissive 2	3	Dominant	1
3	Submissive 1	4	Dominant	0.0037436
3	Submissive 2	4	Dominant	0.152558
3	Submissive 1	5	Dominant	0.0676802
3	Submissive 2	5	Dominant	0.7127227
3	Submissive 1	1	Submissive 1	0.0024468
3	Submissive 2	1	Submissive 1	0.1140887
3	Submissive 1	2	Submissive 1	0.0384508
3	Submissive 2	2	Submissive 1	0.5710209
3	Submissive 2	3	Submissive 1	0.9940161
3	Submissive 2	4	Submissive 1	1
3	Submissive 2	5	Submissive 1	0.9997526
3	Submissive 2	1	Submissive 2	0.0085128
3	Submissive 2	2	Submissive 2	0.9629896
4	Dominant	1	Dominant	0.9999523
4	Submissive 1	1	Dominant	0.8906026
4	Submissive 2	1	Dominant	0.0002583
4	Dominant	2	Dominant	1
4	Submissive 1	2	Dominant	0.600063

Journal of Experimental Biology • Supplementary information

4	Submissive 2	2	Dominant	0.000038
4	Dominant	3	Dominant	0.1140887
4	Submissive 1	3	Dominant	0.9999995
4	Submissive 2	3	Dominant	0.3252194
4	Submissive 1	4	Dominant	0.3751263
4	Submissive 2	4	Dominant	0.0000101
4	Submissive 1	5	Dominant	0.9333776
4	Submissive 2	5	Dominant	0.0004109
4	Submissive 1	1	Submissive 1	0.3017179
4	Submissive 2	1	Submissive 1	0.0000061
4	Submissive 1	2	Submissive 1	0.8543768
4	Submissive 2	2	Submissive 1	0.0001888
4	Submissive 1	3	Submissive 1	0.9206487
4	Submissive 2	3	Submissive 1	0.9629896
4	Submissive 2	4	Submissive 1	0.0930923
4	Submissive 2	5	Submissive 1	0.0237815
4	Submissive 2	1	Submissive 2	0.0000001
4	Submissive 2	2	Submissive 2	0.0032521
4	Submissive 2	3	Submissive 2	0.2578589
5	Dominant	1	Dominant	1
5	Submissive 1	1	Dominant	0.9918766
5	Submissive 2	1	Dominant	0.0110909
5	Dominant	2	Dominant	0.9999987
5	Submissive 1	2	Dominant	0.8906026
5	Submissive 2	2	Dominant	0.0021192
5	Dominant	3	Dominant	0.6289186
5	Submissive 1	3	Dominant	0.9990263
5	Submissive 2	3	Dominant	0.9446316
5	Dominant	4	Dominant	0.9997526
5	Submissive 1	4	Dominant	0.7127227
5	Submissive 2	4	Dominant	0.0006493
5	Submissive 1	5	Dominant	0.9969232
5	Submissive 2	5	Dominant	0.0163383
5	Submissive 1	1	Submissive 1	0.6289186
5	Submissive 2	1	Submissive 1	0.0004109
5	Submissive 1	2	Submissive 1	0.9857418
5	Submissive 2	2	Submissive 1	0.0085128
5	Submissive 1	3	Submissive 1	0.6574231
5	Submissive 2	3	Submissive 1	0.9999999
5	Submissive 1	4	Submissive 1	0.9999999
5	Submissive 2	4	Submissive 1	0.6574231
5	Submissive 2	5	Submissive 1	0.3252194
5	Submissive 2	1	Submissive 2	0.000012

Journal of Experimental Biology • Supplementary information

5	Submissive 2	2	Submissive 2	0.0838572
5	Submissive 2	3	Submissive 2	0.906399
5	Submissive 2	4	Submissive 2	0.9990263
C3 chamber 1				
1	Submissive 1	1	Dominant	0.9932497
1	Submissive 2	1	Dominant	0.7554828
1	Submissive 1	2	Dominant	0.0077176
1	Submissive 2	2	Dominant	0.0005006
1	Submissive 1	3	Dominant	0.18339
1	Submissive 2	3	Dominant	0.0238609
1	Submissive 1	4	Dominant	0.0069945
1	Submissive 2	4	Dominant	0.0715788
1	Submissive 1	5	Dominant	0.1049083
1	Submissive 2	5	Dominant	0.4884815
1	Submissive 2	1	Submissive 1	0.9999747
1	Submissive 2	2	Submissive 1	0.0902951
1	Submissive 2	3	Submissive 1	0.0051878
1	Submissive 2	4	Submissive 1	0.0001611
1	Submissive 2	5	Submissive 1	0.0004477
2	Dominant	1	Dominant	0.250738
2	Submissive 1	1	Dominant	0.995772
2	Submissive 2	1	Dominant	0.4074099
2	Submissive 1	2	Dominant	0.9505375
2	Submissive 2	2	Dominant	1
2	Submissive 1	3	Dominant	1
2	Submissive 2	3	Dominant	0.999906
2	Submissive 1	4	Dominant	0.0000004
2	Submissive 2	4	Dominant	0
2	Submissive 1	5	Dominant	0.0000192
2	Submissive 2	5	Dominant	0.0000001
2	Submissive 1	1	Submissive 1	0.4473253
2	Submissive 2	1	Submissive 1	0.0181654
2	Submissive 2	2	Submissive 1	0.9896105
2	Submissive 2	3	Submissive 1	1
2	Submissive 2	4	Submissive 1	0.9999995
2	Submissive 2	5	Submissive 1	1
2	Submissive 2	1	Submissive 2	0.0013409
3	Dominant	1	Dominant	0.925833
3	Submissive 1	1	Dominant	0.677472
3	Submissive 2	1	Dominant	0.9967029
3	Dominant	2	Dominant	0.9980579
3	Submissive 1	2	Dominant	0.9999969
3	Submissive 2	2	Dominant	0.9430684

Journal of Experimental Biology • Supplementary information

3	Submissive 1	3	Dominant	1
3	Submissive 2	3	Dominant	0.9999999
3	Submissive 1	4	Dominant	0
3	Submissive 2	4	Dominant	0.0000005
3	Submissive 1	5	Dominant	0.0000004
3	Submissive 2	5	Dominant	0.0000217
3	Submissive 1	1	Submissive 1	0.0562686
3	Submissive 2	1	Submissive 1	0.4677716
3	Submissive 1	2	Submissive 1	0.9997048
3	Submissive 2	2	Submissive 1	1
3	Submissive 2	3	Submissive 1	0.9995816
3	Submissive 2	4	Submissive 1	0.8244502
3	Submissive 2	5	Submissive 1	0.9348419
3	Submissive 2	1	Submissive 2	0.097376
3	Submissive 2	2	Submissive 2	0.9872743
4	Dominant	1	Dominant	0.0000634
4	Submissive 1	1	Dominant	0.1303873
4	Submissive 2	1	Dominant	0.0218036
4	Dominant	2	Dominant	0
4	Submissive 1	2	Dominant	1
4	Submissive 2	2	Dominant	0.9997048
4	Dominant	3	Dominant	0.0000001
4	Submissive 1	3	Dominant	0.9813581
4	Submissive 2	3	Dominant	0.7366961
4	Submissive 1	4	Dominant	0
4	Submissive 2	4	Dominant	0
4	Submissive 1	5	Dominant	0
4	Submissive 2	5	Dominant	0
4	Submissive 1	1	Submissive 1	0.0028096
4	Submissive 2	1	Submissive 1	0.0002547
4	Submissive 1	2	Submissive 1	0.8399321
4	Submissive 2	2	Submissive 1	0.4074099
4	Submissive 1	3	Submissive 1	0.9997048
4	Submissive 2	3	Submissive 1	0.9505375
4	Submissive 2	4	Submissive 1	0.9999969
4	Submissive 2	5	Submissive 1	0.999795
4	Submissive 2	1	Submissive 2	0.0000118
4	Submissive 2	2	Submissive 2	0.995772
4	Submissive 2	3	Submissive 2	0.3880251
5	Dominant	1	Dominant	0.0020525
5	Submissive 1	1	Dominant	0.2360895
5	Submissive 2	1	Dominant	0.5516677
5	Dominant	2	Dominant	0

5	Submissive 1	2	Dominant	1
5	Submissive 2	2	Dominant	1
5	Dominant	3	Dominant	0.0000027
5	Submissive 1	3	Dominant	0.9974556
5	Submissive 2	3	Dominant	0.9999969
5	Dominant	4	Dominant	0.999795
5	Submissive 1	4	Dominant	0
5	Submissive 2	4	Dominant	0
5	Submissive 1	5	Dominant	0
5	Submissive 2	5	Dominant	0.0000002
5	Submissive 1	1	Submissive 1	0.0069945
5	Submissive 2	1	Submissive 1	0.0339605
5	Submissive 1	2	Submissive 1	0.9430684
5	Submissive 2	2	Submissive 1	0.9980579
5	Submissive 1	3	Submissive 1	0.9999945
5	Submissive 2	3	Submissive 1	1
5	Submissive 1	4	Submissive 1	1
5	Submissive 2	4	Submissive 1	0.9999747
5	Submissive 2	5	Submissive 1	0.9999999
5	Submissive 2	1	Submissive 2	0.0028096
5	Submissive 2	2	Submissive 2	1
5	Submissive 2	3	Submissive 2	0.9974556
5	Submissive 2	4	Submissive 2	0.9813581
C4 chamber 1				
1	Submissive 1	1	Dominant	0.999736
1	Submissive 2	1	Dominant	0.7648894
1	Submissive 1	2	Dominant	1
1	Submissive 2	2	Dominant	0.9996212
1	Submissive 1	3	Dominant	1
1	Submissive 2	3	Dominant	0.9998191
1	Submissive 1	4	Dominant	0.9992555
1	Submissive 2	4	Dominant	1
1	Submissive 1	5	Dominant	1
1	Submissive 2	5	Dominant	0.9999877
1	Submissive 2	1	Submissive 1	0.998617
1	Submissive 2	2	Submissive 1	1
1	Submissive 2	3	Submissive 1	0.9625642
1	Submissive 2	4	Submissive 1	0.9999997
1	Submissive 2	5	Submissive 1	0.9998191
2	Dominant	1	Dominant	0.9989784
2	Submissive 1	1	Dominant	0.6227315
2	Submissive 2	1	Dominant	0.999736
2	Submissive 1	2	Dominant	0.9968135

Journal of Experimental Biology • Supplementary information

2	Submissive 2	2	Dominant	1
2	Submissive 1	3	Dominant	0.9981515
2	Submissive 2	3	Dominant	1
2	Submissive 1	4	Dominant	1
2	Submissive 2	4	Dominant	0.9992555
2	Submissive 1	5	Dominant	0.999736
2	Submissive 2	5	Dominant	1
2	Submissive 1	1	Submissive 1	0.9916704
2	Submissive 2	1	Submissive 1	1
2	Submissive 2	2	Submissive 1	0.9916704
2	Submissive 2	3	Submissive 1	1
2	Submissive 2	4	Submissive 1	0.9999997
2	Submissive 2	5	Submissive 1	1
2	Submissive 2	1	Submissive 2	0.998617
3	Dominant	1	Dominant	0.9981515
3	Submissive 1	1	Dominant	0.9999999
3	Submissive 2	1	Dominant	0.9730949
3	Dominant	2	Dominant	1
3	Submissive 1	2	Dominant	0.9999994
3	Submissive 2	2	Dominant	1
3	Submissive 1	3	Dominant	0.9999977
3	Submissive 2	3	Dominant	1
3	Submissive 1	4	Dominant	0.9730949
3	Submissive 2	4	Dominant	0.9999999
3	Submissive 1	5	Dominant	0.9999479
3	Submissive 2	5	Dominant	1
3	Submissive 1	1	Submissive 1	1
3	Submissive 2	1	Submissive 1	0.9999997
3	Submissive 1	2	Submissive 1	0.9021817
3	Submissive 2	2	Submissive 1	0.9999796
3	Submissive 2	3	Submissive 1	0.999465
3	Submissive 2	4	Submissive 1	1
3	Submissive 2	5	Submissive 1	1
3	Submissive 2	1	Submissive 2	0.9999997
3	Submissive 2	2	Submissive 2	0.9999997
4	Dominant	1	Dominant	0.8008059
4	Submissive 1	1	Dominant	0.9730949
4	Submissive 2	1	Dominant	0.9999994
4	Dominant	2	Dominant	0.9998191
4	Submissive 1	2	Dominant	1
4	Submissive 2	2	Dominant	0.9414922
4	Dominant	3	Dominant	0.9999195
4	Submissive 1	3	Dominant	1

Journal of Experimental Biology • Supplementary information

4	Submissive 2	3	Dominant	0.9235189
4	Submissive 1	4	Dominant	0.9999999
4	Submissive 2	4	Dominant	0.4100544
4	Submissive 1	5	Dominant	1
4	Submissive 2	5	Dominant	0.8491935
4	Submissive 1	1	Submissive 1	0.9999997
4	Submissive 2	1	Submissive 1	0.9681542
4	Submissive 1	2	Submissive 1	0.9999796
4	Submissive 2	2	Submissive 1	0.2497676
4	Submissive 1	3	Submissive 1	0.999465
4	Submissive 2	3	Submissive 1	0.9989784
4	Submissive 2	4	Submissive 1	0.7459711
4	Submissive 2	5	Submissive 1	0.9235189
4	Submissive 2	1	Submissive 2	0.370767
4	Submissive 2	2	Submissive 2	0.9681542
4	Submissive 2	3	Submissive 2	0.7459711
5	Dominant	1	Dominant	0.9916704
5	Submissive 1	1	Dominant	0.9981515
5	Submissive 2	1	Dominant	0.9730949
5	Dominant	2	Dominant	1
5	Submissive 1	2	Dominant	1
5	Submissive 2	2	Dominant	1
5	Dominant	3	Dominant	1
5	Submissive 1	3	Dominant	1
5	Submissive 2	3	Dominant	1
5	Dominant	4	Dominant	0.9999959
5	Submissive 1	4	Dominant	0.9999195
5	Submissive 2	4	Dominant	0.9999999
5	Submissive 1	5	Dominant	1
5	Submissive 2	5	Dominant	1
5	Submissive 1	1	Submissive 1	1
5	Submissive 2	1	Submissive 1	0.9999997
5	Submissive 1	2	Submissive 1	0.9981515
5	Submissive 2	2	Submissive 1	0.9999796
5	Submissive 1	3	Submissive 1	0.9999977
5	Submissive 2	3	Submissive 1	0.999465
5	Submissive 1	4	Submissive 1	1
5	Submissive 2	4	Submissive 1	1
5	Submissive 2	5	Submissive 1	1
5	Submissive 2	1	Submissive 2	0.9999997
5	Submissive 2	2	Submissive 2	0.9999997
5	Submissive 2	3	Submissive 2	1
5	Submissive 2	4	Submissive 2	0.7459711

Journal of Experimental Biology • Supplementary information

C2 chamber 2				
1	Submissive 1	1	Dominant	0.9999647
1	Submissive 2	1	Dominant	0.9992589
1	Submissive 1	2	Dominant	0.6945744
1	Submissive 2	2	Dominant	0.837873
1	Submissive 1	3	Dominant	0.0313279
1	Submissive 2	3	Dominant	0.0614676
1	Submissive 1	4	Dominant	0.8756885
1	Submissive 2	4	Dominant	0.7465228
1	Submissive 1	5	Dominant	0.1972474
1	Submissive 2	5	Dominant	0.113793
1	Submissive 2	1	Submissive 1	1
1	Submissive 2	2	Submissive 1	0.3655814
1	Submissive 2	3	Submissive 1	0.2733101
1	Submissive 2	4	Submissive 1	0.0194205
1	Submissive 2	5	Submissive 1	0.021931
2	Dominant	1	Dominant	0.1972474
2	Submissive 1	1	Dominant	0.0313279
2	Submissive 2	1	Dominant	0.0117899
2	Submissive 1	2	Dominant	0.9999904
2	Submissive 2	2	Dominant	0.9992589
2	Submissive 1	3	Dominant	0.9999647
2	Submissive 2	3	Dominant	0.9999999
2	Submissive 1	4	Dominant	0.0010051
2	Submissive 2	4	Dominant	0.0003069
2	Submissive 1	5	Dominant	0.0000132
2	Submissive 2	5	Dominant	0.0000036
2	Submissive 1	1	Submissive 1	0.233176
2	Submissive 2	1	Submissive 1	0.113793
2	Submissive 2	2	Submissive 1	1
2	Submissive 2	3	Submissive 1	1
2	Submissive 2	4	Submissive 1	0.9998897
2	Submissive 2	5	Submissive 1	0.9999365
2	Submissive 2	1	Submissive 2	0.1972474
3	Dominant	1	Dominant	0.0023653
3	Submissive 1	1	Dominant	0.0194205
3	Submissive 2	1	Dominant	0.1253169
3	Dominant	2	Dominant	0.9698476
3	Submissive 1	2	Dominant	0.9998897
3	Submissive 2	2	Dominant	1
3	Submissive 1	3	Dominant	0.9999978
3	Submissive 2	3	Dominant	0.991418
3	Submissive 1	4	Dominant	0.0005587

Journal of Experimental Biology • Supplementary information

3	Submissive 2	4	Dominant	0.0061508
3	Submissive 1	5	Dominant	0.0000069
3	Submissive 2	5	Dominant	0.0001048
3	Submissive 1	1	Submissive 1	0.1654895
3	Submissive 2	1	Submissive 1	0.5551962
3	Submissive 1	2	Submissive 1	1
3	Submissive 2	2	Submissive 1	0.9999999
3	Submissive 2	3	Submissive 1	0.9999953
3	Submissive 2	4	Submissive 1	0.9214687
3	Submissive 2	5	Submissive 1	0.9338121
3	Submissive 2	1	Submissive 2	0.7209675
3	Submissive 2	2	Submissive 2	0.9999365
4	Dominant	1	Dominant	0.9992589
4	Submissive 1	1	Dominant	0.0005587
4	Submissive 2	1	Dominant	0.0000132
4	Dominant	2	Dominant	0.0117899
4	Submissive 1	2	Dominant	0.837873
4	Submissive 2	2	Dominant	0.233176
4	Dominant	3	Dominant	0.0000477
4	Submissive 1	3	Dominant	1
4	Submissive 2	3	Dominant	0.9886532
4	Submissive 1	4	Dominant	0.0000095
4	Submissive 2	4	Dominant	0.0000002
4	Submissive 1	5	Dominant	0.0000001
4	Submissive 2	5	Dominant	0
4	Submissive 1	1	Submissive 1	0.0091194
4	Submissive 2	1	Submissive 1	0.0003069
4	Submissive 1	2	Submissive 1	0.9966312
4	Submissive 2	2	Submissive 1	0.6945744
4	Submissive 1	3	Submissive 1	0.9992589
4	Submissive 2	3	Submissive 1	0.7945957
4	Submissive 2	4	Submissive 1	0.9996976
4	Submissive 2	5	Submissive 1	0.9995204
4	Submissive 2	1	Submissive 2	0.0007506
4	Submissive 2	2	Submissive 2	0.8756885
4	Submissive 2	3	Submissive 2	0.3411085
5	Dominant	1	Dominant	0.6945744
5	Submissive 1	1	Dominant	0.0006478
5	Submissive 2	1	Dominant	0.0053823
5	Dominant	2	Dominant	0.0002265
5	Submissive 1	2	Dominant	0.8574936
5	Submissive 2	2	Dominant	0.9936095
5	Dominant	3	Dominant	0.0000005

Journal of Experimental Biology • Supplementary information

5	Submissive 1	3	Dominant	1
5	Submissive 2	3	Dominant	1
5	Dominant	4	Dominant	0.9983516
5	Submissive 1	4	Dominant	0.0000112
5	Submissive 2	4	Dominant	0.0001224
5	Submissive 1	5	Dominant	0.0000001
5	Submissive 2	5	Dominant	0.0000013
5	Submissive 1	1	Submissive 1	0.0103751
5	Submissive 2	1	Submissive 1	0.0614676
5	Submissive 1	2	Submissive 1	0.9976201
5	Submissive 2	2	Submissive 1	0.9999996
5	Submissive 1	3	Submissive 1	0.9995204
5	Submissive 2	3	Submissive 1	1
5	Submissive 1	4	Submissive 1	1
5	Submissive 2	4	Submissive 1	0.9999978
5	Submissive 2	5	Submissive 1	0.999999
5	Submissive 2	1	Submissive 2	0.113793
5	Submissive 2	2	Submissive 2	1
5	Submissive 2	3	Submissive 2	0.9988822
5	Submissive 2	4	Submissive 2	0.9543563
C3 chamber 2				
1	Submissive 1	1	Dominant	1
1	Submissive 2	1	Dominant	0.9999999
1	Submissive 1	2	Dominant	0.8115239
1	Submissive 2	2	Dominant	0.8415427
1	Submissive 1	3	Dominant	0.6880881
1	Submissive 2	3	Dominant	0.7257835
1	Submissive 1	4	Dominant	0.9142597
1	Submissive 2	4	Dominant	0.8930064
1	Submissive 1	5	Dominant	0.5687014
1	Submissive 2	5	Dominant	0.5282718
1	Submissive 2	1	Submissive 1	1
1	Submissive 2	2	Submissive 1	0.8415427
1	Submissive 2	3	Submissive 1	0.9325403
1	Submissive 2	4	Submissive 1	1
1	Submissive 2	5	Submissive 1	0.9999991
2	Dominant	1	Dominant	0.5282718
2	Submissive 1	1	Dominant	0.5282718
2	Submissive 2	1	Dominant	0.0638591
2	Submissive 1	2	Dominant	1
2	Submissive 2	2	Dominant	0.9993498
2	Submissive 1	3	Dominant	1
2	Submissive 2	3	Dominant	0.9999466

Journal of Experimental Biology • Supplementary information

2	Submissive 1	4	Dominant	0.0285824
2	Submissive 2	4	Dominant	0.000938
2	Submissive 1	5	Dominant	0.0042392
2	Submissive 2	5	Dominant	0.0000975
2	Submissive 1	1	Submissive 1	0.8115239
2	Submissive 2	1	Submissive 1	0.1818237
2	Submissive 2	2	Submissive 1	0.9993498
2	Submissive 2	3	Submissive 1	0.9946826
2	Submissive 2	4	Submissive 1	0.0800177
2	Submissive 2	5	Submissive 1	0.04673
2	Submissive 2	1	Submissive 2	0.205632
3	Dominant	1	Dominant	0.3922519
3	Submissive 1	1	Dominant	0.6880881
3	Submissive 2	1	Dominant	0.9899908
3	Dominant	2	Dominant	1
3	Submissive 1	2	Dominant	1
3	Submissive 2	2	Dominant	0.9991215
3	Submissive 1	3	Dominant	1
3	Submissive 2	3	Dominant	0.9946826
3	Submissive 1	4	Dominant	0.0547151
3	Submissive 2	4	Dominant	0.3563121
3	Submissive 1	5	Dominant	0.0090489
3	Submissive 2	5	Dominant	0.0994868
3	Submissive 1	1	Submissive 1	0.9142597
3	Submissive 2	1	Submissive 1	0.9998291
3	Submissive 1	2	Submissive 1	1
3	Submissive 2	2	Submissive 1	0.9991215
3	Submissive 2	3	Submissive 1	0.9999466
3	Submissive 2	4	Submissive 1	0.9946826
3	Submissive 2	5	Submissive 1	0.9790885
3	Submissive 2	1	Submissive 2	0.99992
3	Submissive 2	2	Submissive 2	0.7439967
4	Dominant	1	Dominant	0.9918273
4	Submissive 1	1	Dominant	1
4	Submissive 2	1	Dominant	0.9999997
4	Dominant	2	Dominant	0.0285824
4	Submissive 1	2	Dominant	0.5889338
4	Submissive 2	2	Dominant	0.8687496
4	Dominant	3	Dominant	0.0155959
4	Submissive 1	3	Dominant	0.448966
4	Submissive 2	3	Dominant	0.7617213
4	Submissive 1	4	Dominant	0.9853251
4	Submissive 2	4	Dominant	0.8687496

Journal of Experimental Biology • Supplementary information

4	Submissive 1	5	Dominant	0.7955253
4	Submissive 2	5	Dominant	0.4882323
4	Submissive 1	1	Submissive 1	1
4	Submissive 2	1	Submissive 1	1
4	Submissive 1	2	Submissive 1	0.5889338
4	Submissive 2	2	Submissive 1	0.8687496
4	Submissive 1	3	Submissive 1	0.7439967
4	Submissive 2	3	Submissive 1	0.9479579
4	Submissive 2	4	Submissive 1	1
4	Submissive 2	5	Submissive 1	0.999997
4	Submissive 2	1	Submissive 2	1
4	Submissive 2	2	Submissive 2	0.2315777
4	Submissive 2	3	Submissive 2	0.9999651
5	Dominant	1	Dominant	0.8415427
5	Submissive 1	1	Dominant	1
5	Submissive 2	1	Dominant	0.9996572
5	Dominant	2	Dominant	0.0042392
5	Submissive 1	2	Dominant	0.448966
5	Submissive 2	2	Dominant	0.0742848
5	Dominant	3	Dominant	0.0021244
5	Submissive 1	3	Dominant	0.3221314
5	Submissive 2	3	Dominant	0.0431351
5	Dominant	4	Dominant	0.9999991
5	Submissive 1	4	Dominant	0.9966588
5	Submissive 2	4	Dominant	1
5	Submissive 1	5	Dominant	0.8930064
5	Submissive 2	5	Dominant	0.9997562
5	Submissive 1	1	Submissive 1	0.9999997
5	Submissive 2	1	Submissive 1	0.9853251
5	Submissive 1	2	Submissive 1	0.448966
5	Submissive 2	2	Submissive 1	0.0742848
5	Submissive 1	3	Submissive 1	0.6091062
5	Submissive 2	3	Submissive 1	0.1313236
5	Submissive 1	4	Submissive 1	1
5	Submissive 2	4	Submissive 1	0.9991215
5	Submissive 2	5	Submissive 1	0.99992
5	Submissive 2	1	Submissive 2	0.9790885
5	Submissive 2	2	Submissive 2	0.0031621
5	Submissive 2	3	Submissive 2	0.5889338
5	Submissive 2	4	Submissive 2	0.9709768
C4 chamber 2				
1	Submissive 1	1	Dominant	1
1	Submissive 2	1	Dominant	1

Journal of Experimental Biology • Supplementary information

1	Submissive 1	2	Dominant	0.9999979
1	Submissive 2	2	Dominant	1
1	Submissive 1	3	Dominant	0.9998992
1	Submissive 2	3	Dominant	0.9999979
1	Submissive 1	4	Dominant	0.9872735
1	Submissive 2	4	Dominant	0.9515659
1	Submissive 1	5	Dominant	1
1	Submissive 2	5	Dominant	1
1	Submissive 2	1	Submissive 1	1
1	Submissive 2	2	Submissive 1	1
1	Submissive 2	3	Submissive 1	0.997978
1	Submissive 2	4	Submissive 1	0.992021
1	Submissive 2	5	Submissive 1	1
2	Dominant	1	Dominant	0.9999368
2	Submissive 1	1	Dominant	0.9999979
2	Submissive 2	1	Dominant	0.997978
2	Submissive 1	2	Dominant	1
2	Submissive 2	2	Dominant	0.8049687
2	Submissive 1	3	Dominant	1
2	Submissive 2	3	Dominant	0.6559561
2	Submissive 1	4	Dominant	0.8868471
2	Submissive 2	4	Dominant	1
2	Submissive 1	5	Dominant	1
2	Submissive 2	5	Dominant	0.9124804
2	Submissive 1	1	Submissive 1	1
2	Submissive 2	1	Submissive 1	0.9898694
2	Submissive 2	2	Submissive 1	0.9001841
2	Submissive 2	3	Submissive 1	1
2	Submissive 2	4	Submissive 1	1
2	Submissive 2	5	Submissive 1	0.9339941
2	Submissive 2	1	Submissive 2	0.9589657
3	Dominant	1	Dominant	0.998939
3	Submissive 1	1	Dominant	0.9999925
3	Submissive 2	1	Dominant	0.9841738
3	Dominant	2	Dominant	1
3	Submissive 1	2	Dominant	0.9655042
3	Submissive 2	2	Dominant	0.9999995
3	Submissive 1	3	Dominant	0.9001841
3	Submissive 2	3	Dominant	1
3	Submissive 1	4	Dominant	1
3	Submissive 2	4	Dominant	0.3997833
3	Submissive 1	5	Dominant	0.992021
3	Submissive 2	5	Dominant	0.9999613

Journal of Experimental Biology • Supplementary information

3	Submissive 1	1	Submissive 1	0.9998431
3	Submissive 2	1	Submissive 1	0.9963622
3	Submissive 1	2	Submissive 1	0.9898694
3	Submissive 2	2	Submissive 1	0.999977
3	Submissive 2	3	Submissive 1	0.7235559
3	Submissive 2	4	Submissive 1	0.6090417
3	Submissive 2	5	Submissive 1	0.9998992
3	Submissive 2	1	Submissive 2	0.9996434
3	Submissive 2	2	Submissive 2	0.4218338
4	Dominant	1	Dominant	0.9972693
4	Submissive 1	1	Dominant	0.9998992
4	Submissive 2	1	Dominant	1
4	Dominant	2	Dominant	0.7857775
4	Submissive 1	2	Dominant	0.9237445
4	Submissive 2	2	Dominant	0.9963622
4	Dominant	3	Dominant	0.6326238
4	Submissive 1	3	Dominant	0.82328
4	Submissive 2	3	Dominant	0.9805089
4	Submissive 1	4	Dominant	1
4	Submissive 2	4	Dominant	0.9999613
4	Submissive 1	5	Dominant	0.9762167
4	Submissive 2	5	Dominant	0.9996434
4	Submissive 1	1	Submissive 1	0.998939
4	Submissive 2	1	Submissive 1	0.9999995
4	Submissive 1	2	Submissive 1	0.9712353
4	Submissive 2	2	Submissive 1	0.9994779
4	Submissive 1	3	Submissive 1	1
4	Submissive 2	3	Submissive 1	1
4	Submissive 2	4	Submissive 1	0.9999999
4	Submissive 2	5	Submissive 1	0.9998431
4	Submissive 2	1	Submissive 2	0.9999613
4	Submissive 2	2	Submissive 2	0.999977
4	Submissive 2	3	Submissive 2	0.9001841
5	Dominant	1	Dominant	0.999999
5	Submissive 1	1	Dominant	0.9999998
5	Submissive 2	1	Dominant	0.9999613
5	Dominant	2	Dominant	1
5	Submissive 1	2	Dominant	1
5	Submissive 2	2	Dominant	1
5	Dominant	3	Dominant	1
5	Submissive 1	3	Dominant	0.9999998
5	Submissive 2	3	Dominant	1
5	Dominant	4	Dominant	0.9001841

Journal of Experimental Biology • Supplementary information

5	Submissive 1	4	Dominant	0.9237445
5	Submissive 2	4	Dominant	0.8049687
5	Submissive 1	5	Dominant	1
5	Submissive 2	5	Dominant	1
5	Submissive 1	1	Submissive 1	1
5	Submissive 2	1	Submissive 1	0.999999
5	Submissive 1	2	Submissive 1	1
5	Submissive 2	2	Submissive 1	1
5	Submissive 1	3	Submissive 1	0.9952159
5	Submissive 2	3	Submissive 1	0.9712353
5	Submissive 1	4	Submissive 1	0.9841738
5	Submissive 2	4	Submissive 1	0.9339941
5	Submissive 2	5	Submissive 1	1
5	Submissive 2	1	Submissive 2	1
5	Submissive 2	2	Submissive 2	0.82328
5	Submissive 2	3	Submissive 2	0.999999
5	Submissive 2	4	Submissive 2	0.9972693

Table S3. p-values of the comparisons in heart rate.

Experimental day	Subject	Experimental day	Subject	p-value
C				
2	Dominant	1	Dominant	0.9999999
3	Dominant	1	Dominant	1
4	Dominant	1	Dominant	1
5	Dominant	1	Dominant	0.9999981
1	Submissive 1	1	Dominant	0.9974247
2	Submissive 1	1	Dominant	1
3	Submissive 1	1	Dominant	0.9999981
4	Submissive 1	1	Dominant	0.9993836
5	Submissive 1	1	Dominant	1
1	Submissive 2	1	Dominant	1
2	Submissive 2	1	Dominant	1
3	Submissive 2	1	Dominant	0.9974247
4	Submissive 2	1	Dominant	1
5	Submissive 2	1	Dominant	0.9983487
3	Dominant	2	Dominant	0.9999744
4	Dominant	2	Dominant	0.9999981
5	Dominant	2	Dominant	1
1	Submissive 1	2	Dominant	0.9999951
2	Submissive 1	2	Dominant	0.9999981
3	Submissive 1	2	Dominant	1
4	Submissive 1	2	Dominant	0.9714924

Journal of Experimental Biology • Supplementary information

5	Submissive 1	2	Dominant	0.9998945
1	Submissive 2	2	Dominant	1
2	Submissive 2	2	Dominant	0.9999993
3	Submissive 2	2	Dominant	0.9999951
4	Submissive 2	2	Dominant	1
5	Submissive 2	2	Dominant	0.9527185
4	Dominant	3	Dominant	1
5	Dominant	3	Dominant	0.9998016
1	Submissive 1	3	Dominant	0.9784379
2	Submissive 1	3	Dominant	1
3	Submissive 1	3	Dominant	0.9998016
4	Submissive 1	3	Dominant	0.9999884
5	Submissive 1	3	Dominant	1
1	Submissive 2	3	Dominant	1
2	Submissive 2	3	Dominant	1
3	Submissive 2	3	Dominant	0.9784379
4	Submissive 2	3	Dominant	1
5	Submissive 2	3	Dominant	0.9999466
5	Dominant	4	Dominant	0.9999744
1	Submissive 1	4	Dominant	0.9917213
2	Submissive 1	4	Dominant	1
3	Submissive 1	4	Dominant	0.9999744
4	Submissive 1	4	Dominant	0.9998945
5	Submissive 1	4	Dominant	1
1	Submissive 2	4	Dominant	1
2	Submissive 2	4	Dominant	1
3	Submissive 2	4	Dominant	0.9917213
4	Submissive 2	4	Dominant	1
5	Submissive 2	4	Dominant	0.9996432
1	Submissive 1	5	Dominant	0.9999998
2	Submissive 1	5	Dominant	0.9999744
3	Submissive 1	5	Dominant	1
4	Submissive 1	5	Dominant	0.940562
5	Submissive 1	5	Dominant	0.9993836
1	Submissive 2	5	Dominant	0.9999998
2	Submissive 2	5	Dominant	0.9999884
3	Submissive 2	5	Dominant	0.9999998
4	Submissive 2	5	Dominant	0.9999999
5	Submissive 2	5	Dominant	0.91008
2	Submissive 1	1	Submissive 1	0.9917213
3	Submissive 1	1	Submissive 1	0.9999998
4	Submissive 1	1	Submissive 1	0.6731791
5	Submissive 1	1	Submissive 1	0.9629772

Journal of Experimental Biology • Supplementary information

1	Submissive 2	1	Submissive 1	0.9989739
2	Submissive 2	1	Submissive 1	0.9942451
3	Submissive 2	1	Submissive 1	1
4	Submissive 2	1	Submissive 1	0.9993836
5	Submissive 2	1	Submissive 1	0.605426
3	Submissive 1	2	Submissive 1	0.9999744
4	Submissive 1	2	Submissive 1	0.9998945
5	Submissive 1	2	Submissive 1	1
1	Submissive 2	2	Submissive 1	1
2	Submissive 2	2	Submissive 1	1
3	Submissive 2	2	Submissive 1	0.9917213
4	Submissive 2	2	Submissive 1	1
5	Submissive 2	2	Submissive 1	0.9996432
4	Submissive 1	3	Submissive 1	0.940562
5	Submissive 1	3	Submissive 1	0.9993836
1	Submissive 2	3	Submissive 1	0.9999998
2	Submissive 2	3	Submissive 1	0.9999884
3	Submissive 2	3	Submissive 1	0.9999998
4	Submissive 2	3	Submissive 1	0.9999999
5	Submissive 2	3	Submissive 1	0.91008
5	Submissive 1	4	Submissive 1	0.9999981
1	Submissive 2	4	Submissive 1	0.9983487
2	Submissive 2	4	Submissive 1	0.9998016
3	Submissive 2	4	Submissive 1	0.6731791
4	Submissive 2	4	Submissive 1	0.9974247
5	Submissive 2	4	Submissive 1	1
1	Submissive 2	5	Submissive 1	0.9999999
2	Submissive 2	5	Submissive 1	1
3	Submissive 2	5	Submissive 1	0.9629772
4	Submissive 2	5	Submissive 1	0.9999998
5	Submissive 2	5	Submissive 1	0.9999884
2	Submissive 2	1	Submissive 2	1
3	Submissive 2	1	Submissive 2	0.9989739
4	Submissive 2	1	Submissive 2	1
5	Submissive 2	1	Submissive 2	0.9960982
3	Submissive 2	2	Submissive 2	0.9942451
4	Submissive 2	2	Submissive 2	1
5	Submissive 2	2	Submissive 2	0.9993836
4	Submissive 2	3	Submissive 2	0.9993836
5	Submissive 2	3	Submissive 2	0.605426
5	Submissive 2	4	Submissive 2	0.9942451
C2				
1	Submissive 1	1	Dominant	1

Journal of Experimental Biology • Supplementary information

1	Submissive 2	1	Dominant	0.9999947
1	Submissive 1	2	Dominant	0.9999843
1	Submissive 2	2	Dominant	0.9995297
1	Submissive 1	3	Dominant	0.0022666
1	Submissive 2	3	Dominant	0.0009288
1	Submissive 1	4	Dominant	0
1	Submissive 2	4	Dominant	0
1	Submissive 1	5	Dominant	0.0000002
1	Submissive 2	5	Dominant	0.0000007
1	Submissive 2	1	Submissive 1	1
1	Submissive 2	2	Submissive 1	0.7596149
1	Submissive 2	3	Submissive 1	0.0000877
1	Submissive 2	4	Submissive 1	0
1	Submissive 2	5	Submissive 1	0.0002914
2	Dominant	1	Dominant	1
2	Submissive 1	1	Dominant	0.9876476
2	Submissive 2	1	Dominant	0.9999999
2	Submissive 1	2	Dominant	0.9990811
2	Submissive 2	2	Dominant	1
2	Submissive 1	3	Dominant	0.3394168
2	Submissive 2	3	Dominant	0.0446443
2	Submissive 1	4	Dominant	0
2	Submissive 2	4	Dominant	0
2	Submissive 1	5	Dominant	0
2	Submissive 2	5	Dominant	0
2	Submissive 1	1	Submissive 1	0.8886455
2	Submissive 2	1	Submissive 1	0.9997741
2	Submissive 2	2	Submissive 1	0.999899
2	Submissive 2	3	Submissive 1	0.0065681
2	Submissive 2	4	Submissive 1	0.0000001
2	Submissive 2	5	Submissive 1	0.0178085
2	Submissive 2	1	Submissive 2	0.9970251
3	Dominant	1	Dominant	0.009873
3	Submissive 1	1	Dominant	0.0011645
3	Submissive 2	1	Dominant	0.0053356
3	Dominant	2	Dominant	0.0259829
3	Submissive 1	2	Dominant	0.0034944
3	Submissive 2	2	Dominant	0.0146735
3	Submissive 1	3	Dominant	0.9999984
3	Submissive 2	3	Dominant	1
3	Submissive 1	4	Dominant	0
3	Submissive 2	4	Dominant	0
3	Submissive 1	5	Dominant	0

Journal of Experimental Biology • Supplementary information

3	Submissive 2	5	Dominant	0
3	Submissive 1	1	Submissive 1	0.0002299
3	Submissive 2	1	Submissive 1	0.0011645
3	Submissive 1	2	Submissive 1	0.0871951
3	Submissive 2	2	Submissive 1	0.2379669
3	Submissive 2	3	Submissive 1	1
3	Submissive 2	4	Submissive 1	0.0742006
3	Submissive 2	5	Submissive 1	1
3	Submissive 2	1	Submissive 2	0.0004658
3	Submissive 2	2	Submissive 2	0.0259829
4	Dominant	1	Dominant	0
4	Submissive 1	1	Dominant	0
4	Submissive 2	1	Dominant	0.0000198
4	Dominant	2	Dominant	0
4	Submissive 1	2	Dominant	0
4	Submissive 2	2	Dominant	0.0000686
4	Dominant	3	Dominant	0
4	Submissive 1	3	Dominant	0.0446443
4	Submissive 2	3	Dominant	0.9324764
4	Submissive 1	4	Dominant	0
4	Submissive 2	4	Dominant	0
4	Submissive 1	5	Dominant	0
4	Submissive 2	5	Dominant	0
4	Submissive 1	1	Submissive 1	0
4	Submissive 2	1	Submissive 1	0.0000033
4	Submissive 1	2	Submissive 1	0.0000026
4	Submissive 2	2	Submissive 1	0.0034944
4	Submissive 1	3	Submissive 1	0.209174
4	Submissive 2	3	Submissive 1	0.9990811
4	Submissive 2	4	Submissive 1	0.8613717
4	Submissive 2	5	Submissive 1	0.9876476
4	Submissive 2	1	Submissive 2	0.0000012
4	Submissive 2	2	Submissive 2	0.0001424
4	Submissive 2	3	Submissive 2	0.9734067
5	Dominant	1	Dominant	0
5	Submissive 1	1	Dominant	0.0034944
5	Submissive 2	1	Dominant	0.0000026
5	Dominant	2	Dominant	0
5	Submissive 1	2	Dominant	0.009873
5	Submissive 2	2	Dominant	0.0000093
5	Dominant	3	Dominant	0
5	Submissive 1	3	Dominant	1
5	Submissive 2	3	Dominant	0.6786058

Journal of Experimental Biology • Supplementary information

5	Dominant	4	Dominant	0.7201227
5	Submissive 1	4	Dominant	0
5	Submissive 2	4	Dominant	0
5	Submissive 1	5	Dominant	0
5	Submissive 2	5	Dominant	0
5	Submissive 1	1	Submissive 1	0.0007393
5	Submissive 2	1	Submissive 1	0.0000004
5	Submissive 1	2	Submissive 1	0.1829366
5	Submissive 2	2	Submissive 1	0.0005874
5	Submissive 1	3	Submissive 1	1
5	Submissive 2	3	Submissive 1	0.9627346
5	Submissive 1	4	Submissive 1	0.1020351
5	Submissive 2	4	Submissive 1	0.9876476
5	Submissive 2	5	Submissive 1	0.8613717
5	Submissive 2	1	Submissive 2	0.0000001
5	Submissive 2	2	Submissive 2	0.0000198
5	Submissive 2	3	Submissive 2	0.7965856
5	Submissive 2	4	Submissive 2	0.9999999
C3				
1	Submissive 1	1	Dominant	0.9983266
1	Submissive 2	1	Dominant	0.9838177
1	Submissive 1	2	Dominant	0.9941738
1	Submissive 2	2	Dominant	0.962598
1	Submissive 1	3	Dominant	0.9998929
1	Submissive 2	3	Dominant	0.997391
1	Submissive 1	4	Dominant	0
1	Submissive 2	4	Dominant	0
1	Submissive 1	5	Dominant	0
1	Submissive 2	5	Dominant	0
1	Submissive 2	1	Submissive 1	1
1	Submissive 2	2	Submissive 1	0.3398581
1	Submissive 2	3	Submissive 1	1
1	Submissive 2	4	Submissive 1	1
1	Submissive 2	5	Submissive 1	0.9999951
2	Dominant	1	Dominant	1
2	Submissive 1	1	Dominant	0.9941738
2	Submissive 2	1	Dominant	1
2	Submissive 1	2	Dominant	0.9983266
2	Submissive 2	2	Dominant	0.9999999
2	Submissive 1	3	Dominant	0.9711902
2	Submissive 2	3	Dominant	1
2	Submissive 1	4	Dominant	0
2	Submissive 2	4	Dominant	0

Journal of Experimental Biology • Supplementary information

2	Submissive 1	5	Dominant	0
2	Submissive 2	5	Dominant	0
2	Submissive 1	1	Submissive 1	0.5343161
2	Submissive 2	1	Submissive 1	0.999974
2	Submissive 2	2	Submissive 1	0.9522513
2	Submissive 2	3	Submissive 1	0.9941738
2	Submissive 2	4	Submissive 1	0.9996381
2	Submissive 2	5	Submissive 1	0.9092885
2	Submissive 2	1	Submissive 2	0.9989598
3	Dominant	1	Dominant	1
3	Submissive 1	1	Dominant	0.9522513
3	Submissive 2	1	Dominant	1
3	Dominant	2	Dominant	1
3	Submissive 1	2	Dominant	0.9092885
3	Submissive 2	2	Dominant	1
3	Submissive 1	3	Dominant	0.9882288
3	Submissive 2	3	Dominant	0.9999993
3	Submissive 1	4	Dominant	0
3	Submissive 2	4	Dominant	0
3	Submissive 1	5	Dominant	0
3	Submissive 2	5	Dominant	0
3	Submissive 1	1	Submissive 1	0.9999999
3	Submissive 2	1	Submissive 1	0.9782015
3	Submissive 1	2	Submissive 1	0.2345366
3	Submissive 2	2	Submissive 1	0.9997987
3	Submissive 2	3	Submissive 1	0.8219312
3	Submissive 2	4	Submissive 1	0.9399962
3	Submissive 2	5	Submissive 1	0.5000115
3	Submissive 2	1	Submissive 2	0.9092885
3	Submissive 2	2	Submissive 2	0.9999951
4	Dominant	1	Dominant	0
4	Submissive 1	1	Dominant	0.9916216
4	Submissive 2	1	Dominant	0.9999998
4	Dominant	2	Dominant	0
4	Submissive 1	2	Dominant	0.9782015
4	Submissive 2	2	Dominant	0.9999951
4	Dominant	3	Dominant	0
4	Submissive 1	3	Dominant	0.9989598
4	Submissive 2	3	Dominant	1
4	Submissive 1	4	Dominant	0
4	Submissive 2	4	Dominant	0
4	Submissive 1	5	Dominant	0
4	Submissive 2	5	Dominant	0

Journal of Experimental Biology • Supplementary information

4	Submissive 1	1	Submissive 1	1
4	Submissive 2	1	Submissive 1	0.9999993
4	Submissive 1	2	Submissive 1	0.4008761
4	Submissive 2	2	Submissive 1	0.8906899
4	Submissive 1	3	Submissive 1	1
4	Submissive 2	3	Submissive 1	0.9989598
4	Submissive 2	4	Submissive 1	0.999974
4	Submissive 2	5	Submissive 1	0.962598
4	Submissive 2	1	Submissive 2	0.9998929
4	Submissive 2	2	Submissive 2	1
4	Submissive 2	3	Submissive 2	0.9998929
5	Dominant	1	Dominant	0
5	Submissive 1	1	Dominant	0.7358358
5	Submissive 2	1	Dominant	0.9882288
5	Dominant	2	Dominant	0
5	Submissive 1	2	Dominant	0.6376244
5	Submissive 2	2	Dominant	0.9711902
5	Dominant	3	Dominant	0
5	Submissive 1	3	Dominant	0.8699001
5	Submissive 2	3	Dominant	0.9983266
5	Dominant	4	Dominant	0.9999993
5	Submissive 1	4	Dominant	0
5	Submissive 2	4	Dominant	0
5	Submissive 1	5	Dominant	0
5	Submissive 2	5	Dominant	0
5	Submissive 1	1	Submissive 1	0.9996381
5	Submissive 2	1	Submissive 1	1
5	Submissive 1	2	Submissive 1	0.0750456
5	Submissive 2	2	Submissive 1	0.3697532
5	Submissive 1	3	Submissive 1	0.9999999
5	Submissive 2	3	Submissive 1	1
5	Submissive 1	4	Submissive 1	0.999974
5	Submissive 2	4	Submissive 1	1
5	Submissive 2	5	Submissive 1	0.9999883
5	Submissive 2	1	Submissive 2	1
5	Submissive 2	2	Submissive 2	0.999375
5	Submissive 2	3	Submissive 2	0.9257062
5	Submissive 2	4	Submissive 2	0.9999458
C4				
1	Submissive 1	1	Dominant	0.9988582
1	Submissive 2	1	Dominant	0.3260835
1	Submissive 1	2	Dominant	0.999601
1	Submissive 2	2	Dominant	0.9999978

Journal of Experimental Biology • Supplementary information

1	Submissive 1	3	Dominant	1
1	Submissive 2	3	Dominant	0.9956924
1	Submissive 1	4	Dominant	1
1	Submissive 2	4	Dominant	0.8625825
1	Submissive 1	5	Dominant	1
1	Submissive 2	5	Dominant	0.9489493
1	Submissive 2	1	Submissive 1	0.9489493
1	Submissive 2	2	Submissive 1	0.4514651
1	Submissive 2	3	Submissive 1	1
1	Submissive 2	4	Submissive 1	0.8842649
1	Submissive 2	5	Submissive 1	0.7241689
2	Dominant	1	Dominant	0.7553203
2	Submissive 1	1	Dominant	1
2	Submissive 2	1	Dominant	1
2	Submissive 1	2	Dominant	0.8625825
2	Submissive 2	2	Dominant	0.6917255
2	Submissive 1	3	Dominant	0.9936637
2	Submissive 2	3	Dominant	0.9599129
2	Submissive 1	4	Dominant	0.9999978
2	Submissive 2	4	Dominant	0.9997777
2	Submissive 1	5	Dominant	0.9998815
2	Submissive 2	5	Dominant	0.9971493
2	Submissive 1	1	Submissive 1	0.9998815
2	Submissive 2	1	Submissive 1	0.9971493
2	Submissive 2	2	Submissive 1	1
2	Submissive 2	3	Submissive 1	0.4852915
2	Submissive 2	4	Submissive 1	0.999601
2	Submissive 2	5	Submissive 1	0.9999945
2	Submissive 2	1	Submissive 2	0.2713673
3	Dominant	1	Dominant	0.9765214
3	Submissive 1	1	Dominant	0.5544859
3	Submissive 2	1	Dominant	1
3	Dominant	2	Dominant	0.9999992
3	Submissive 1	2	Dominant	1
3	Submissive 2	2	Dominant	0.8842649
3	Submissive 1	3	Dominant	0.9998815
3	Submissive 2	3	Dominant	0.9956924
3	Submissive 1	4	Dominant	0.9690458
3	Submissive 2	4	Dominant	0.9999992
3	Submissive 1	5	Dominant	0.9936637
3	Submissive 2	5	Dominant	0.9999399
3	Submissive 1	1	Submissive 1	0.9936637
3	Submissive 2	1	Submissive 1	0.9999399

Journal of Experimental Biology • Supplementary information

3	Submissive 1	2	Submissive 1	0.6917255
3	Submissive 2	2	Submissive 1	1
3	Submissive 2	3	Submissive 1	0.7241689
3	Submissive 2	4	Submissive 1	0.9999978
3	Submissive 2	5	Submissive 1	1
3	Submissive 2	1	Submissive 2	0.4852915
3	Submissive 2	2	Submissive 2	1
4	Dominant	1	Dominant	0.9999399
4	Submissive 1	1	Dominant	0.9998815
4	Submissive 2	1	Dominant	0.999601
4	Dominant	2	Dominant	0.9956924
4	Submissive 1	2	Dominant	0.9971493
4	Submissive 2	2	Dominant	0.9988582
4	Dominant	3	Dominant	0.9999978
4	Submissive 1	3	Dominant	0.9999992
4	Submissive 2	3	Dominant	0.9999999
4	Submissive 1	4	Dominant	1
4	Submissive 2	4	Dominant	1
4	Submissive 1	5	Dominant	1
4	Submissive 2	5	Dominant	1
4	Submissive 1	1	Submissive 1	1
4	Submissive 2	1	Submissive 1	1
4	Submissive 1	2	Submissive 1	0.9999945
4	Submissive 2	2	Submissive 1	0.9999711
4	Submissive 1	3	Submissive 1	0.9765214
4	Submissive 2	3	Submissive 1	0.9872595
4	Submissive 2	4	Submissive 1	1
4	Submissive 2	5	Submissive 1	1
4	Submissive 2	1	Submissive 2	0.9209569
4	Submissive 2	2	Submissive 2	0.9988582
4	Submissive 2	3	Submissive 2	0.9999869
5	Dominant	1	Dominant	0.9988582
5	Submissive 1	1	Dominant	0.9999992
5	Submissive 2	1	Dominant	0.9999711
5	Dominant	2	Dominant	0.999601
5	Submissive 1	2	Dominant	0.9765214
5	Submissive 2	2	Dominant	0.9936637
5	Dominant	3	Dominant	1
5	Submissive 1	3	Dominant	0.9998815
5	Submissive 2	3	Dominant	0.9999945
5	Dominant	4	Dominant	1
5	Submissive 1	4	Dominant	1
5	Submissive 2	4	Dominant	1

Journal of Experimental Biology • Supplementary information

5	Submissive 1	5	Dominant	0.9999999
5	Submissive 2	5	Dominant	1
5	Submissive 1	1	Submissive 1	0.9999999
5	Submissive 2	1	Submissive 1	1
5	Submissive 1	2	Submissive 1	1
5	Submissive 2	2	Submissive 1	0.9999992
5	Submissive 1	3	Submissive 1	0.9037244
5	Submissive 2	3	Submissive 1	0.9599129
5	Submissive 1	4	Submissive 1	1
5	Submissive 2	4	Submissive 1	1
5	Submissive 2	5	Submissive 1	1
5	Submissive 2	1	Submissive 2	0.838728
5	Submissive 2	2	Submissive 2	0.9998815
5	Submissive 2	3	Submissive 2	0.9999998
5	Submissive 2	4	Submissive 2	1

Table S4. p-values of the comparisons in heart rate.

Experimental day	Subject	Experimental day	Subject	p-value
C chamber 1				
1	Submissive 1	1	Dominant	0.9950498
1	Submissive 2	1	Dominant	0.430092
1	Submissive 1	2	Dominant	0.999994
1	Submissive 2	2	Dominant	0.999999
1	Submissive 1	3	Dominant	0.9985229
1	Submissive 2	3	Dominant	1
1	Submissive 1	4	Dominant	1
1	Submissive 2	4	Dominant	0.9865386
1	Submissive 1	5	Dominant	0.999999
1	Submissive 2	5	Dominant	0.8509599
1	Submissive 2	1	Submissive 1	0.9929429
1	Submissive 2	2	Submissive 1	0.9999439
1	Submissive 2	3	Submissive 1	0.8025296
1	Submissive 2	4	Submissive 1	0.9505479
1	Submissive 2	5	Submissive 1	0.9998057
2	Dominant	1	Dominant	0.8276347
2	Submissive 1	1	Dominant	0.9250384
2	Submissive 2	1	Dominant	0.9999975
2	Submissive 1	2	Dominant	1
2	Submissive 2	2	Dominant	0.9929429
2	Submissive 1	3	Dominant	0.9999975
2	Submissive 2	3	Dominant	0.9250384
2	Submissive 1	4	Dominant	0.9999997

2	Submissive 2	4	Dominant	0.9999999
2	Submissive 1	5	Dominant	0.9990655
2	Submissive 2	5	Dominant	1
2	Submissive 1	1	Submissive 1	1
2	Submissive 2	1	Submissive 1	0.999999
2	Submissive 2	2	Submissive 1	0.9990655
2	Submissive 2	3	Submissive 1	1
2	Submissive 2	4	Submissive 1	1
2	Submissive 2	5	Submissive 1	0.9996601
2	Submissive 2	1	Submissive 2	0.8509599
3	Dominant	1	Dominant	0.5583823
3	Submissive 1	1	Dominant	0.9999997
3	Submissive 2	1	Dominant	0.9094545
3	Dominant	2	Dominant	1
3	Submissive 1	2	Dominant	0.9865386
3	Submissive 2	2	Dominant	1
3	Submissive 1	3	Dominant	0.8919141
3	Submissive 2	3	Dominant	0.999999
3	Submissive 1	4	Dominant	0.999999
3	Submissive 2	4	Dominant	0.999999
3	Submissive 1	5	Dominant	1
3	Submissive 2	5	Dominant	0.9985229
3	Submissive 1	1	Submissive 1	0.999994
3	Submissive 2	1	Submissive 1	0.9999999
3	Submissive 1	2	Submissive 1	0.9977313
3	Submissive 2	2	Submissive 1	1
3	Submissive 2	3	Submissive 1	0.996607
3	Submissive 2	4	Submissive 1	0.9999439
3	Submissive 2	5	Submissive 1	1
3	Submissive 2	1	Submissive 2	0.9999719
3	Submissive 2	2	Submissive 2	0.9985229
4	Dominant	1	Dominant	0.9977313
4	Submissive 1	1	Dominant	0.9998057
4	Submissive 2	1	Dominant	1
4	Dominant	2	Dominant	0.9999719
4	Submissive 1	2	Dominant	0.9994269
4	Submissive 2	2	Dominant	0.8276347
4	Dominant	3	Dominant	0.996607
4	Submissive 1	3	Dominant	0.981941
4	Submissive 2	3	Dominant	0.5583823
4	Submissive 1	4	Dominant	1
4	Submissive 2	4	Dominant	0.9977313
4	Submissive 1	5	Dominant	1

Journal of Experimental Biology • Supplementary information

4	Submissive 2	5	Dominant	0.9999975
4	Submissive 1	1	Submissive 1	1
4	Submissive 2	1	Submissive 1	0.9950498
4	Submissive 1	2	Submissive 1	0.9999719
4	Submissive 2	2	Submissive 1	0.9250384
4	Submissive 1	3	Submissive 1	1
4	Submissive 2	3	Submissive 1	0.9999997
4	Submissive 2	4	Submissive 1	0.9998057
4	Submissive 2	5	Submissive 1	0.9505479
4	Submissive 2	1	Submissive 2	0.430092
4	Submissive 2	2	Submissive 2	0.9999975
4	Submissive 2	3	Submissive 2	0.9094545
5	Dominant	1	Dominant	0.9999975
5	Submissive 1	1	Dominant	0.9505479
5	Submissive 2	1	Dominant	0.8276347
5	Dominant	2	Dominant	0.9929429
5	Submissive 1	2	Dominant	1
5	Submissive 2	2	Dominant	1
5	Dominant	3	Dominant	0.9250384
5	Submissive 1	3	Dominant	0.9999867
5	Submissive 2	3	Dominant	1
5	Dominant	4	Dominant	0.9999999
5	Submissive 1	4	Dominant	1
5	Submissive 2	4	Dominant	0.9999719
5	Submissive 1	5	Dominant	0.9996601
5	Submissive 2	5	Dominant	0.9929429
5	Submissive 1	1	Submissive 1	1
5	Submissive 2	1	Submissive 1	0.999994
5	Submissive 1	2	Submissive 1	1
5	Submissive 2	2	Submissive 1	1
5	Submissive 1	3	Submissive 1	0.9990655
5	Submissive 2	3	Submissive 1	0.9865386
5	Submissive 1	4	Submissive 1	0.999994
5	Submissive 2	4	Submissive 1	0.9994269
5	Submissive 2	5	Submissive 1	1
5	Submissive 2	1	Submissive 2	0.999999
5	Submissive 2	2	Submissive 2	0.9929429
5	Submissive 2	3	Submissive 2	1
5	Submissive 2	4	Submissive 2	0.8276347
C2 chamber 1				
1	Submissive 1	1	Dominant	1
1	Submissive 2	1	Dominant	0.9999933
1	Submissive 1	2	Dominant	0.8848429

Journal of Experimental Biology • Supplementary information

1	Submissive 2	2	Dominant	0.9853226
1	Submissive 1	3	Dominant	0
1	Submissive 2	3	Dominant	0.0000001
1	Submissive 1	4	Dominant	1
1	Submissive 2	4	Dominant	1
1	Submissive 1	5	Dominant	0.9998382
1	Submissive 2	5	Dominant	0.9999999
1	Submissive 2	1	Submissive 1	1
1	Submissive 2	2	Submissive 1	0.9117554
1	Submissive 2	3	Submissive 1	0.0000036
1	Submissive 2	4	Submissive 1	0.0235049
1	Submissive 2	5	Submissive 1	0.0122934
2	Dominant	1	Dominant	0.7349229
2	Submissive 1	1	Dominant	0.4931703
2	Submissive 2	1	Dominant	0.6890211
2	Submissive 1	2	Dominant	1
2	Submissive 2	2	Dominant	1
2	Submissive 1	3	Dominant	0.0001829
2	Submissive 2	3	Dominant	0.0000616
2	Submissive 1	4	Dominant	0.7349229
2	Submissive 2	4	Dominant	0.8848429
2	Submissive 1	5	Dominant	0.9945617
2	Submissive 2	5	Dominant	0.9996213
2	Submissive 1	1	Submissive 1	0.6890211
2	Submissive 2	1	Submissive 1	0.8534421
2	Submissive 2	2	Submissive 1	1
2	Submissive 2	3	Submissive 1	0.0011357
2	Submissive 2	4	Submissive 1	0.5919341
2	Submissive 2	5	Submissive 1	0.4450633
2	Submissive 2	1	Submissive 2	0.977434
3	Dominant	1	Dominant	0
3	Submissive 1	1	Dominant	0.0000002
3	Submissive 2	1	Dominant	0.0000008
3	Dominant	2	Dominant	0.0000467
3	Submissive 1	2	Dominant	0.000881
3	Submissive 2	2	Dominant	0.003054
3	Submissive 1	3	Dominant	0.9999781
3	Submissive 2	3	Dominant	0.9983654
3	Submissive 1	4	Dominant	0.0000008
3	Submissive 2	4	Dominant	0.0000036
3	Submissive 1	5	Dominant	0.0000267
3	Submissive 2	5	Dominant	0.0001065
3	Submissive 1	1	Submissive 1	0.0000006

Journal of Experimental Biology • Supplementary information

3	Submissive 2	1	Submissive 1	0.0000027
3	Submissive 1	2	Submissive 1	0.003054
3	Submissive 2	2	Submissive 1	0.0098301
3	Submissive 2	3	Submissive 1	1
3	Submissive 2	4	Submissive 1	0.7780428
3	Submissive 2	5	Submissive 1	0.8848429
3	Submissive 2	1	Submissive 2	0.0000152
3	Submissive 2	2	Submissive 2	0.0038831
4	Dominant	1	Dominant	1
4	Submissive 1	1	Dominant	0.0023949
4	Submissive 2	1	Dominant	0
4	Dominant	2	Dominant	0.9117554
4	Submissive 1	2	Dominant	0.5423667
4	Submissive 2	2	Dominant	0.0000006
4	Dominant	3	Dominant	0
4	Submissive 1	3	Dominant	0.1291032
4	Submissive 2	3	Dominant	0.999184
4	Submissive 1	4	Dominant	0.0078324
4	Submissive 2	4	Dominant	0
4	Submissive 1	5	Dominant	0.0915907
4	Submissive 2	5	Dominant	0
4	Submissive 1	1	Submissive 1	0.0062193
4	Submissive 2	1	Submissive 1	0
4	Submissive 1	2	Submissive 1	0.7780428
4	Submissive 2	2	Submissive 1	0.0000027
4	Submissive 1	3	Submissive 1	0.5423667
4	Submissive 2	3	Submissive 1	0.8848429
4	Submissive 2	4	Submissive 1	0.0062193
4	Submissive 2	5	Submissive 1	0.0122934
4	Submissive 2	1	Submissive 2	0
4	Submissive 2	2	Submissive 2	0.0000008
4	Submissive 2	3	Submissive 2	0.6890211
5	Dominant	1	Dominant	0.9969309
5	Submissive 1	1	Dominant	0.0011357
5	Submissive 2	1	Dominant	0
5	Dominant	2	Dominant	0.9998382
5	Submissive 1	2	Dominant	0.3986751
5	Submissive 2	2	Dominant	0
5	Dominant	3	Dominant	0.0000011
5	Submissive 1	3	Dominant	0.2068541
5	Submissive 2	3	Dominant	0.6890211
5	Dominant	4	Dominant	0.9999371
5	Submissive 1	4	Dominant	0.0038831

5	Submissive 2	4	Dominant	0
5	Submissive 1	5	Dominant	0.0526054
5	Submissive 2	5	Dominant	0
5	Submissive 1	1	Submissive 1	0.003054
5	Submissive 2	1	Submissive 1	0
5	Submissive 1	2	Submissive 1	0.64109
5	Submissive 2	2	Submissive 1	0
5	Submissive 1	3	Submissive 1	0.6890211
5	Submissive 2	3	Submissive 1	0.2068541
5	Submissive 1	4	Submissive 1	1
5	Submissive 2	4	Submissive 1	0.0001065
5	Submissive 2	5	Submissive 1	0.0002389
5	Submissive 2	1	Submissive 2	0
5	Submissive 2	2	Submissive 2	0
5	Submissive 2	3	Submissive 2	0.0915907
5	Submissive 2	4	Submissive 2	0.9983654
C3 chamber 1				
1	Submissive 1	1	Dominant	0.9999852
1	Submissive 2	1	Dominant	0.9999972
1	Submissive 1	2	Dominant	0.0000001
1	Submissive 2	2	Dominant	0.0000001
1	Submissive 1	3	Dominant	0.0000171
1	Submissive 2	3	Dominant	0.0000249
1	Submissive 1	4	Dominant	0.0000006
1	Submissive 2	4	Dominant	0.0000004
1	Submissive 1	5	Dominant	0.00003
1	Submissive 2	5	Dominant	0.0000206
1	Submissive 2	1	Submissive 1	1
1	Submissive 2	2	Submissive 1	0.0046114
1	Submissive 2	3	Submissive 1	0.0014828
1	Submissive 2	4	Submissive 1	0.000001
1	Submissive 2	5	Submissive 1	0.0000003
2	Dominant	1	Dominant	0.0000014
2	Submissive 1	1	Dominant	0.0358246
2	Submissive 2	1	Dominant	0.0012547
2	Submissive 1	2	Dominant	0.3012754
2	Submissive 2	2	Dominant	0.9202567
2	Submissive 1	3	Dominant	0.9854451
2	Submissive 2	3	Dominant	1
2	Submissive 1	4	Dominant	0
2	Submissive 2	4	Dominant	0
2	Submissive 1	5	Dominant	0
2	Submissive 2	5	Dominant	0

Journal of Experimental Biology • Supplementary information

2	Submissive 1	1	Submissive 1	0.0033564
2	Submissive 2	1	Submissive 1	0.0000754
2	Submissive 2	2	Submissive 1	0.9993693
2	Submissive 2	3	Submissive 1	0.9999934
2	Submissive 2	4	Submissive 1	0.9975193
2	Submissive 2	5	Submissive 1	0.9805191
2	Submissive 2	1	Submissive 2	0.0001086
3	Dominant	1	Dominant	0.0003171
3	Submissive 1	1	Dominant	0.0133885
3	Submissive 2	1	Dominant	0.0358246
3	Dominant	2	Dominant	0.98933
3	Submissive 1	2	Dominant	0.5110635
3	Submissive 2	2	Dominant	0.3012754
3	Submissive 1	3	Dominant	0.9989738
3	Submissive 2	3	Dominant	0.9854451
3	Submissive 1	4	Dominant	0
3	Submissive 2	4	Dominant	0
3	Submissive 1	5	Dominant	0
3	Submissive 2	5	Dominant	0
3	Submissive 1	1	Submissive 1	0.0010605
3	Submissive 2	1	Submissive 1	0.0033564
3	Submissive 1	2	Submissive 1	1
3	Submissive 2	2	Submissive 1	1
3	Submissive 2	3	Submissive 1	1
3	Submissive 2	4	Submissive 1	0.6746178
3	Submissive 2	5	Submissive 1	0.4785688
3	Submissive 2	1	Submissive 2	0.0046114
3	Submissive 2	2	Submissive 2	0.9993693
4	Dominant	1	Dominant	0
4	Submissive 1	1	Dominant	0.0000141
4	Submissive 2	1	Dominant	0.0000097
4	Dominant	2	Dominant	0
4	Submissive 1	2	Dominant	0.9999996
4	Submissive 2	2	Dominant	1
4	Dominant	3	Dominant	0
4	Submissive 1	3	Dominant	0.9999688
4	Submissive 2	3	Dominant	0.9998819
4	Submissive 1	4	Dominant	0
4	Submissive 2	4	Dominant	0
4	Submissive 1	5	Dominant	0
4	Submissive 2	5	Dominant	0
4	Submissive 1	1	Submissive 1	0.0000006
4	Submissive 2	1	Submissive 1	0.0000004

Journal of Experimental Biology • Supplementary information

4	Submissive 1	2	Submissive 1	0.6746178
4	Submissive 2	2	Submissive 1	0.6099014
4	Submissive 1	3	Submissive 1	0.8651962
4	Submissive 2	3	Submissive 1	0.8187046
4	Submissive 2	4	Submissive 1	1
4	Submissive 2	5	Submissive 1	1
4	Submissive 2	1	Submissive 2	0.0000006
4	Submissive 2	2	Submissive 2	0.9946111
4	Submissive 2	3	Submissive 2	0.6099014
5	Dominant	1	Dominant	0.0000014
5	Submissive 1	1	Dominant	0.0000045
5	Submissive 2	1	Dominant	0.00003
5	Dominant	2	Dominant	0
5	Submissive 1	2	Dominant	1
5	Submissive 2	2	Dominant	0.9999852
5	Dominant	3	Dominant	0
5	Submissive 1	3	Dominant	0.9989738
5	Submissive 2	3	Dominant	0.9999989
5	Dominant	4	Dominant	0.9997853
5	Submissive 1	4	Dominant	0
5	Submissive 2	4	Dominant	0
5	Submissive 1	5	Dominant	0
5	Submissive 2	5	Dominant	0
5	Submissive 1	1	Submissive 1	0.0000002
5	Submissive 2	1	Submissive 1	0.0000014
5	Submissive 1	2	Submissive 1	0.4785688
5	Submissive 2	2	Submissive 1	0.7927532
5	Submissive 1	3	Submissive 1	0.7059048
5	Submissive 2	3	Submissive 1	0.93465
5	Submissive 1	4	Submissive 1	1
5	Submissive 2	4	Submissive 1	1
5	Submissive 2	5	Submissive 1	1
5	Submissive 2	1	Submissive 2	0.0000021
5	Submissive 2	2	Submissive 2	0.9996252
5	Submissive 2	3	Submissive 2	0.7927532
5	Submissive 2	4	Submissive 2	1
C4 chamber 1				
1	Submissive 1	1	Dominant	1
1	Submissive 2	1	Dominant	1
1	Submissive 1	2	Dominant	1
1	Submissive 2	2	Dominant	0.9999914
1	Submissive 1	3	Dominant	0.9999957
1	Submissive 2	3	Dominant	0.9996357

Journal of Experimental Biology • Supplementary information

1	Submissive 1	4	Dominant	0.9893165
1	Submissive 2	4	Dominant	0.9991689
1	Submissive 1	5	Dominant	0.9997672
1	Submissive 2	5	Dominant	0.9999979
1	Submissive 2	1	Submissive 1	1
1	Submissive 2	2	Submissive 1	0.9997672
1	Submissive 2	3	Submissive 1	1
1	Submissive 2	4	Submissive 1	0.9999979
1	Submissive 2	5	Submissive 1	1
2	Dominant	1	Dominant	1
2	Submissive 1	1	Dominant	0.9862599
2	Submissive 2	1	Dominant	0.9999979
2	Submissive 1	2	Dominant	0.9417618
2	Submissive 2	2	Dominant	0.9998549
2	Submissive 1	3	Dominant	0.8408134
2	Submissive 2	3	Dominant	0.9975442
2	Submissive 1	4	Dominant	1
2	Submissive 2	4	Dominant	0.9999121
2	Submissive 1	5	Dominant	1
2	Submissive 2	5	Dominant	1
2	Submissive 1	1	Submissive 1	0.9953719
2	Submissive 2	1	Submissive 1	0.9999999
2	Submissive 2	2	Submissive 1	0.9999838
2	Submissive 2	3	Submissive 1	0.9999999
2	Submissive 2	4	Submissive 1	1
2	Submissive 2	5	Submissive 1	0.9999996
2	Submissive 2	1	Submissive 2	1
3	Dominant	1	Dominant	0.9999998
3	Submissive 1	1	Dominant	1
3	Submissive 2	1	Dominant	1
3	Dominant	2	Dominant	1
3	Submissive 1	2	Dominant	1
3	Submissive 2	2	Dominant	0.9999957
3	Submissive 1	3	Dominant	0.9999957
3	Submissive 2	3	Dominant	0.9997672
3	Submissive 1	4	Dominant	0.9893165
3	Submissive 2	4	Dominant	0.9987843
3	Submissive 1	5	Dominant	0.9997672
3	Submissive 2	5	Dominant	0.9999957
3	Submissive 1	1	Submissive 1	1
3	Submissive 2	1	Submissive 1	1
3	Submissive 1	2	Submissive 1	0.9953719
3	Submissive 2	2	Submissive 1	0.9996357

Journal of Experimental Biology • Supplementary information

3	Submissive 2	3	Submissive 1	1
3	Submissive 2	4	Submissive 1	0.9999957
3	Submissive 2	5	Submissive 1	1
3	Submissive 2	1	Submissive 2	1
3	Submissive 2	2	Submissive 2	1
4	Dominant	1	Dominant	0.972812
4	Submissive 1	1	Dominant	0.9987843
4	Submissive 2	1	Dominant	0.9999914
4	Dominant	2	Dominant	0.9062027
4	Submissive 1	2	Dominant	0.9893165
4	Submissive 2	2	Dominant	1
4	Dominant	3	Dominant	0.7789956
4	Submissive 1	3	Dominant	0.951163
4	Submissive 2	3	Dominant	1
4	Submissive 1	4	Dominant	1
4	Submissive 2	4	Dominant	0.6575612
4	Submissive 1	5	Dominant	1
4	Submissive 2	5	Dominant	0.8917881
4	Submissive 1	1	Submissive 1	0.9997672
4	Submissive 2	1	Submissive 1	0.9999121
4	Submissive 1	2	Submissive 1	1
4	Submissive 2	2	Submissive 1	0.7326772
4	Submissive 1	3	Submissive 1	0.9997672
4	Submissive 2	3	Submissive 1	0.9999121
4	Submissive 2	4	Submissive 1	0.8917881
4	Submissive 2	5	Submissive 1	0.9999706
4	Submissive 2	1	Submissive 2	0.9975442
4	Submissive 2	2	Submissive 2	0.9893165
4	Submissive 2	3	Submissive 2	0.9982562
5	Dominant	1	Dominant	0.9987843
5	Submissive 1	1	Dominant	1
5	Submissive 2	1	Dominant	1
5	Dominant	2	Dominant	0.9893165
5	Submissive 1	2	Dominant	1
5	Submissive 2	2	Dominant	0.9999998
5	Dominant	3	Dominant	0.951163
5	Submissive 1	3	Dominant	0.999999
5	Submissive 2	3	Dominant	0.9999706
5	Dominant	4	Dominant	1
5	Submissive 1	4	Dominant	0.9825479
5	Submissive 2	4	Dominant	0.9953719
5	Submissive 1	5	Dominant	0.9994436
5	Submissive 2	5	Dominant	0.9999483

Journal of Experimental Biology • Supplementary information

5	Submissive 1	1	Submissive 1	1
5	Submissive 2	1	Submissive 1	1
5	Submissive 1	2	Submissive 1	0.9918014
5	Submissive 2	2	Submissive 1	0.9982562
5	Submissive 1	3	Submissive 1	1
5	Submissive 2	3	Submissive 1	1
5	Submissive 1	4	Submissive 1	0.9994436
5	Submissive 2	4	Submissive 1	0.9999483
5	Submissive 2	5	Submissive 1	1
5	Submissive 2	1	Submissive 2	1
5	Submissive 2	2	Submissive 2	1
5	Submissive 2	3	Submissive 2	1
5	Submissive 2	4	Submissive 2	0.9996357
C chamber 2				
1	Submissive 1	1	Dominant	0.9999814
1	Submissive 2	1	Dominant	1
1	Submissive 1	2	Dominant	0.9990616
1	Submissive 2	2	Dominant	0.9999814
1	Submissive 1	3	Dominant	1
1	Submissive 2	3	Dominant	1
1	Submissive 1	4	Dominant	0.9999592
1	Submissive 2	4	Dominant	0.9984532
1	Submissive 1	5	Dominant	1
1	Submissive 2	5	Dominant	1
1	Submissive 2	1	Submissive 1	1
1	Submissive 2	2	Submissive 1	0.9999996
1	Submissive 2	3	Submissive 1	1
1	Submissive 2	4	Submissive 1	1
1	Submissive 2	5	Submissive 1	1
2	Dominant	1	Dominant	1
2	Submissive 1	1	Dominant	0.9994515
2	Submissive 2	1	Dominant	0.9701546
2	Submissive 1	2	Dominant	0.9916785
2	Submissive 2	2	Dominant	0.8835025
2	Submissive 1	3	Dominant	1
2	Submissive 2	3	Dominant	0.9998348
2	Submissive 1	4	Dominant	0.9999996
2	Submissive 2	4	Dominant	1
2	Submissive 1	5	Dominant	0.9999969
2	Submissive 2	5	Dominant	0.9975359
2	Submissive 1	1	Submissive 1	1
2	Submissive 2	1	Submissive 1	0.9999814
2	Submissive 2	2	Submissive 1	0.9999999

Journal of Experimental Biology • Supplementary information

2	Submissive 2	3	Submissive 1	0.9975359
2	Submissive 2	4	Submissive 1	0.9999921
2	Submissive 2	5	Submissive 1	0.9984532
2	Submissive 2	1	Submissive 2	0.9990616
3	Dominant	1	Dominant	0.9999989
3	Submissive 1	1	Dominant	1
3	Submissive 2	1	Dominant	1
3	Dominant	2	Dominant	0.9998348
3	Submissive 1	2	Dominant	0.9999969
3	Submissive 2	2	Dominant	1
3	Submissive 1	3	Dominant	1
3	Submissive 2	3	Dominant	0.9990616
3	Submissive 1	4	Dominant	0.9961965
3	Submissive 2	4	Dominant	0.7805361
3	Submissive 1	5	Dominant	1
3	Submissive 2	5	Dominant	0.9999592
3	Submissive 1	1	Submissive 1	1
3	Submissive 2	1	Submissive 1	0.9961965
3	Submissive 1	2	Submissive 1	0.9999969
3	Submissive 2	2	Submissive 1	0.9776062
3	Submissive 2	3	Submissive 1	0.9999592
3	Submissive 2	4	Submissive 1	0.9942976
3	Submissive 2	5	Submissive 1	0.9999156
3	Submissive 2	1	Submissive 2	0.9998348
3	Submissive 2	2	Submissive 2	0.8094616
4	Dominant	1	Dominant	0.9609751
4	Submissive 1	1	Dominant	0.9999592
4	Submissive 2	1	Dominant	0.9999999
4	Dominant	2	Dominant	0.8610792
4	Submissive 1	2	Dominant	0.9984532
4	Submissive 2	2	Dominant	0.9999592
4	Dominant	3	Dominant	0.9996922
4	Submissive 1	3	Dominant	1
4	Submissive 2	3	Dominant	1
4	Submissive 1	4	Dominant	0.9999814
4	Submissive 2	4	Dominant	0.9990616
4	Submissive 1	5	Dominant	1
4	Submissive 2	5	Dominant	1
4	Submissive 1	1	Submissive 1	1
4	Submissive 2	1	Submissive 1	1
4	Submissive 1	2	Submissive 1	1
4	Submissive 2	2	Submissive 1	0.9999999
4	Submissive 1	3	Submissive 1	1

4	Submissive 2	3	Submissive 1	1
4	Submissive 2	4	Submissive 1	1
4	Submissive 2	5	Submissive 1	1
4	Submissive 2	1	Submissive 2	1
4	Submissive 2	2	Submissive 2	0.9994515
4	Submissive 2	3	Submissive 2	0.9996922
5	Dominant	1	Dominant	1
5	Submissive 1	1	Dominant	1
5	Submissive 2	1	Dominant	0.9776062
5	Dominant	2	Dominant	0.9999969
5	Submissive 1	2	Dominant	0.9999921
5	Submissive 2	2	Dominant	0.9975359
5	Dominant	3	Dominant	1
5	Submissive 1	3	Dominant	1
5	Submissive 2	3	Dominant	0.7497888
5	Dominant	4	Dominant	0.9961965
5	Submissive 1	4	Dominant	0.9975359
5	Submissive 2	4	Dominant	0.1668818
5	Submissive 1	5	Dominant	1
5	Submissive 2	5	Dominant	0.8835025
5	Submissive 1	1	Submissive 1	1
5	Submissive 2	1	Submissive 1	0.6491657
5	Submissive 1	2	Submissive 1	0.9999989
5	Submissive 2	2	Submissive 1	0.4712887
5	Submissive 1	3	Submissive 1	1
5	Submissive 2	3	Submissive 1	0.8835025
5	Submissive 1	4	Submissive 1	1
5	Submissive 2	4	Submissive 1	0.6137971
5	Submissive 2	5	Submissive 1	0.8610792
5	Submissive 2	1	Submissive 2	0.8363623
5	Submissive 2	2	Submissive 2	0.1866122
5	Submissive 2	3	Submissive 2	0.9994515
5	Submissive 2	4	Submissive 2	0.8094616
C2 chamber 2				
1	Submissive 1	1	Dominant	1
1	Submissive 2	1	Dominant	0.9995617
1	Submissive 1	2	Dominant	0.0033864
1	Submissive 2	2	Dominant	0.0316697
1	Submissive 1	3	Dominant	0.0000435
1	Submissive 2	3	Dominant	0.0006399
1	Submissive 1	4	Dominant	0.0374874
1	Submissive 2	4	Dominant	0.0041341
1	Submissive 1	5	Dominant	0.0187579

Journal of Experimental Biology • Supplementary information

1	Submissive 2	5	Dominant	0.0018385
1	Submissive 2	1	Submissive 1	0.9999932
1	Submissive 2	2	Submissive 1	0.1111987
1	Submissive 2	3	Submissive 1	0.0061209
1	Submissive 2	4	Submissive 1	0.0001709
1	Submissive 2	5	Submissive 1	0.0000345
2	Dominant	1	Dominant	0.0012118
2	Submissive 1	1	Dominant	0.0061209
2	Submissive 2	1	Dominant	0.0089799
2	Submissive 1	2	Dominant	1
2	Submissive 2	2	Dominant	0.9999993
2	Submissive 1	3	Dominant	0.9463031
2	Submissive 2	3	Dominant	0.9110487
2	Submissive 1	4	Dominant	0
2	Submissive 2	4	Dominant	0
2	Submissive 1	5	Dominant	0
2	Submissive 2	5	Dominant	0
2	Submissive 1	1	Submissive 1	0.0156645
2	Submissive 2	1	Submissive 1	0.0224003
2	Submissive 2	2	Submissive 1	1
2	Submissive 2	3	Submissive 1	0.9985194
2	Submissive 2	4	Submissive 1	0.7333906
2	Submissive 2	5	Submissive 1	0.4510278
2	Submissive 2	1	Submissive 2	0.1467633
3	Dominant	1	Dominant	0.0000135
3	Submissive 1	1	Dominant	0.0001709
3	Submissive 2	1	Dominant	0.0002138
3	Dominant	2	Dominant	0.9974657
3	Submissive 1	2	Dominant	0.9999998
3	Submissive 2	2	Dominant	1
3	Submissive 1	3	Dominant	0.9999977
3	Submissive 2	3	Dominant	0.9999932
3	Submissive 1	4	Dominant	0
3	Submissive 2	4	Dominant	0
3	Submissive 1	5	Dominant	0
3	Submissive 2	5	Dominant	0
3	Submissive 1	1	Submissive 1	0.0005155
3	Submissive 2	1	Submissive 1	0.0006399
3	Submissive 1	2	Submissive 1	0.9995617
3	Submissive 2	2	Submissive 1	0.9997801
3	Submissive 2	3	Submissive 1	1
3	Submissive 2	4	Submissive 1	0.9991738
3	Submissive 2	5	Submissive 1	0.9787264

Journal of Experimental Biology • Supplementary information

3	Submissive 2	1	Submissive 2	0.0074226
3	Submissive 2	2	Submissive 2	0.9991738
4	Dominant	1	Dominant	0.0829586
4	Submissive 1	1	Dominant	0.0000032
4	Submissive 2	1	Dominant	0
4	Dominant	2	Dominant	0
4	Submissive 1	2	Dominant	0.9703075
4	Submissive 2	2	Dominant	0.0962268
4	Dominant	3	Dominant	0
4	Submissive 1	3	Dominant	1
4	Submissive 2	3	Dominant	0.7333906
4	Submissive 1	4	Dominant	0
4	Submissive 2	4	Dominant	0
4	Submissive 1	5	Dominant	0
4	Submissive 2	5	Dominant	0
4	Submissive 1	1	Submissive 1	0.0000106
4	Submissive 2	1	Submissive 1	0
4	Submissive 1	2	Submissive 1	0.8036463
4	Submissive 2	2	Submissive 1	0.026674
4	Submissive 1	3	Submissive 1	0.9995617
4	Submissive 2	3	Submissive 1	0.3046297
4	Submissive 2	4	Submissive 1	0.9110487
4	Submissive 2	5	Submissive 1	0.989965
4	Submissive 2	1	Submissive 2	0.0000001
4	Submissive 2	2	Submissive 2	0.0187579
4	Submissive 2	3	Submissive 2	0.2727954
5	Dominant	1	Dominant	0.0442353
5	Submissive 1	1	Dominant	0.0000006
5	Submissive 2	1	Dominant	0
5	Dominant	2	Dominant	0
5	Submissive 1	2	Dominant	0.8349788
5	Submissive 2	2	Dominant	0.1111987
5	Dominant	3	Dominant	0
5	Submissive 1	3	Dominant	0.9999816
5	Submissive 2	3	Dominant	0.7696741
5	Dominant	4	Dominant	1
5	Submissive 1	4	Dominant	0
5	Submissive 2	4	Dominant	0
5	Submissive 1	5	Dominant	0
5	Submissive 2	5	Dominant	0
5	Submissive 1	1	Submissive 1	0.000002
5	Submissive 2	1	Submissive 1	0
5	Submissive 1	2	Submissive 1	0.5321905

Journal of Experimental Biology • Supplementary information

5	Submissive 2	2	Submissive 1	0.0316697
5	Submissive 1	3	Submissive 1	0.9851707
5	Submissive 2	3	Submissive 1	0.3385491
5	Submissive 1	4	Submissive 1	1
5	Submissive 2	4	Submissive 1	0.9301812
5	Submissive 2	5	Submissive 1	0.9934243
5	Submissive 2	1	Submissive 2	0.0000002
5	Submissive 2	2	Submissive 2	0.0224003
5	Submissive 2	3	Submissive 2	0.3046297
5	Submissive 2	4	Submissive 2	1
C3 chamber 2				
1	Submissive 1	1	Dominant	1
1	Submissive 2	1	Dominant	1
1	Submissive 1	2	Dominant	0.0274892
1	Submissive 2	2	Dominant	0.0696055
1	Submissive 1	3	Dominant	0.0958758
1	Submissive 2	3	Dominant	0.2066224
1	Submissive 1	4	Dominant	0.1175845
1	Submissive 2	4	Dominant	0.0497349
1	Submissive 1	5	Dominant	0.1573461
1	Submissive 2	5	Dominant	0.0696055
1	Submissive 2	1	Submissive 1	1
1	Submissive 2	2	Submissive 1	0.585361
1	Submissive 2	3	Submissive 1	0.4407615
1	Submissive 2	4	Submissive 1	0.9999048
1	Submissive 2	5	Submissive 1	0.9999468
2	Dominant	1	Dominant	0.0274892
2	Submissive 1	1	Dominant	0.3602992
2	Submissive 2	1	Dominant	0.0274892
2	Submissive 1	2	Dominant	0.9989265
2	Submissive 2	2	Dominant	1
2	Submissive 1	3	Dominant	0.9999985
2	Submissive 2	3	Dominant	1
2	Submissive 1	4	Dominant	0.0000135
2	Submissive 2	4	Dominant	0.0000002
2	Submissive 1	5	Dominant	0.0000223
2	Submissive 2	5	Dominant	0.0000003
2	Submissive 1	1	Submissive 1	0.3602992
2	Submissive 2	1	Submissive 1	0.0274892
2	Submissive 2	2	Submissive 1	0.9989265
2	Submissive 2	3	Submissive 1	0.9999048
2	Submissive 2	4	Submissive 1	0.0049915
2	Submissive 2	5	Submissive 1	0.005734

Journal of Experimental Biology • Supplementary information

2	Submissive 2	1	Submissive 2	0.0696055
3	Dominant	1	Dominant	0.0958758
3	Submissive 1	1	Dominant	0.2450567
3	Submissive 2	1	Dominant	0.585361
3	Dominant	2	Dominant	1
3	Submissive 1	2	Dominant	0.9999048
3	Submissive 2	2	Dominant	0.9845369
3	Submissive 1	3	Dominant	1
3	Submissive 2	3	Dominant	0.9997258
3	Submissive 1	4	Dominant	0.0000058
3	Submissive 2	4	Dominant	0.0000509
3	Submissive 1	5	Dominant	0.0000096
3	Submissive 2	5	Dominant	0.000083
3	Submissive 1	1	Submissive 1	0.2450567
3	Submissive 2	1	Submissive 1	0.585361
3	Submissive 1	2	Submissive 1	1
3	Submissive 2	2	Submissive 1	1
3	Submissive 2	3	Submissive 1	0.9999998
3	Submissive 2	4	Submissive 1	0.2450567
3	Submissive 2	5	Submissive 1	0.2659615
3	Submissive 2	1	Submissive 2	0.8023208
3	Submissive 2	2	Submissive 2	0.9845369
4	Dominant	1	Dominant	0.1175845
4	Submissive 1	1	Dominant	0.9999998
4	Submissive 2	1	Dominant	1
4	Dominant	2	Dominant	0.0000002
4	Submissive 1	2	Dominant	0.0049915
4	Submissive 2	2	Dominant	0.0775871
4	Dominant	3	Dominant	0.000001
4	Submissive 1	3	Dominant	0.0214558
4	Submissive 2	3	Dominant	0.2252752
4	Submissive 1	4	Dominant	0.3602992
4	Submissive 2	4	Dominant	0.0443139
4	Submissive 1	5	Dominant	0.4407615
4	Submissive 2	5	Dominant	0.0623346
4	Submissive 1	1	Submissive 1	0.9999998
4	Submissive 2	1	Submissive 1	1
4	Submissive 1	2	Submissive 1	0.1175845
4	Submissive 2	2	Submissive 1	0.6145927
4	Submissive 1	3	Submissive 1	0.0696055
4	Submissive 2	3	Submissive 1	0.4689698
4	Submissive 2	4	Submissive 1	0.9998358
4	Submissive 2	5	Submissive 1	0.9999048

Journal of Experimental Biology • Supplementary information

4	Submissive 2	1	Submissive 2	1
4	Submissive 2	2	Submissive 2	0.0775871
4	Submissive 2	3	Submissive 2	0.8249083
5	Dominant	1	Dominant	0.1573461
5	Submissive 1	1	Dominant	0.9999999
5	Submissive 2	1	Dominant	1
5	Dominant	2	Dominant	0.0000003
5	Submissive 1	2	Dominant	0.005734
5	Submissive 2	2	Dominant	0.0146393
5	Dominant	3	Dominant	0.0000017
5	Submissive 1	3	Dominant	0.0243035
5	Submissive 2	3	Dominant	0.0557267
5	Dominant	4	Dominant	1
5	Submissive 1	4	Dominant	0.33519
5	Submissive 2	4	Dominant	0.1890936
5	Submissive 1	5	Dominant	0.4131752
5	Submissive 2	5	Dominant	0.2450567
5	Submissive 1	1	Submissive 1	0.9999999
5	Submissive 2	1	Submissive 1	1
5	Submissive 1	2	Submissive 1	0.1298368
5	Submissive 2	2	Submissive 1	0.2450567
5	Submissive 1	3	Submissive 1	0.0775871
5	Submissive 2	3	Submissive 1	0.1573461
5	Submissive 1	4	Submissive 1	1
5	Submissive 2	4	Submissive 1	1
5	Submissive 2	5	Submissive 1	1
5	Submissive 2	1	Submissive 2	0.9999998
5	Submissive 2	2	Submissive 2	0.0146393
5	Submissive 2	3	Submissive 2	0.4407615
5	Submissive 2	4	Submissive 2	0.9999994
C4 chamber 2				
1	Submissive 1	1	Dominant	0.9998515
1	Submissive 2	1	Dominant	0.9997456
1	Submissive 1	2	Dominant	1
1	Submissive 2	2	Dominant	1
1	Submissive 1	3	Dominant	0.9995785
1	Submissive 2	3	Dominant	0.9997456
1	Submissive 1	4	Dominant	0.9989418
1	Submissive 2	4	Dominant	0.99839
1	Submissive 1	5	Dominant	1
1	Submissive 2	5	Dominant	1
1	Submissive 2	1	Submissive 1	1
1	Submissive 2	2	Submissive 1	0.9999884

Journal of Experimental Biology • Supplementary information

1	Submissive 2	3	Submissive 1	0.9650829
1	Submissive 2	4	Submissive 1	0.9874154
1	Submissive 2	5	Submissive 1	1
2	Dominant	1	Dominant	0.9999547
2	Submissive 1	1	Dominant	0.9348917
2	Submissive 2	1	Dominant	1
2	Submissive 1	2	Dominant	0.9999164
2	Submissive 2	2	Dominant	0.9995785
2	Submissive 1	3	Dominant	1
2	Submissive 2	3	Dominant	0.7570364
2	Submissive 1	4	Dominant	0.8720802
2	Submissive 2	4	Dominant	1
2	Submissive 1	5	Dominant	0.999999
2	Submissive 2	5	Dominant	0.9950724
2	Submissive 1	1	Submissive 1	0.9999765
2	Submissive 2	1	Submissive 1	0.9989418
2	Submissive 2	2	Submissive 1	0.8720802
2	Submissive 2	3	Submissive 1	1
2	Submissive 2	4	Submissive 1	1
2	Submissive 2	5	Submissive 1	0.9965311
2	Submissive 2	1	Submissive 2	0.99839
3	Dominant	1	Dominant	0.8521544
3	Submissive 1	1	Dominant	0.999999
3	Submissive 2	1	Dominant	0.9833851
3	Dominant	2	Dominant	0.9989418
3	Submissive 1	2	Dominant	0.9833851
3	Submissive 2	2	Dominant	0.999999
3	Submissive 1	3	Dominant	0.4621524
3	Submissive 2	3	Dominant	1
3	Submissive 1	4	Dominant	1
3	Submissive 2	4	Dominant	0.9564898
3	Submissive 1	5	Dominant	0.9348917
3	Submissive 2	5	Dominant	1
3	Submissive 1	1	Submissive 1	0.9723479
3	Submissive 2	1	Submissive 1	0.9999999
3	Submissive 1	2	Submissive 1	0.6132293
3	Submissive 2	2	Submissive 1	1
3	Submissive 2	3	Submissive 1	0.7829262
3	Submissive 2	4	Submissive 1	0.8720802
3	Submissive 2	5	Submissive 1	1
3	Submissive 2	1	Submissive 2	1
3	Submissive 2	2	Submissive 2	0.9564898
4	Dominant	1	Dominant	1

Journal of Experimental Biology • Supplementary information

4	Submissive 1	1	Dominant	1
4	Submissive 2	1	Dominant	1
4	Dominant	2	Dominant	0.9995785
4	Submissive 1	2	Dominant	0.9950724
4	Submissive 2	2	Dominant	0.9999164
4	Dominant	3	Dominant	0.7570364
4	Submissive 1	3	Dominant	0.5828566
4	Submissive 2	3	Dominant	0.8305982
4	Submissive 1	4	Dominant	1
4	Submissive 2	4	Dominant	1
4	Submissive 1	5	Dominant	0.9723479
4	Submissive 2	5	Dominant	0.99839
4	Submissive 1	1	Submissive 1	0.9906255
4	Submissive 2	1	Submissive 1	0.9997456
4	Submissive 1	2	Submissive 1	0.7299632
4	Submissive 2	2	Submissive 1	0.9217138
4	Submissive 1	3	Submissive 1	1
4	Submissive 2	3	Submissive 1	0.9999996
4	Submissive 2	4	Submissive 1	1
4	Submissive 2	5	Submissive 1	0.9989418
4	Submissive 2	1	Submissive 2	0.9995785
4	Submissive 2	2	Submissive 2	1
4	Submissive 2	3	Submissive 2	0.978406
5	Dominant	1	Dominant	0.9989418
5	Submissive 1	1	Dominant	0.9993226
5	Submissive 2	1	Dominant	1
5	Dominant	2	Dominant	1
5	Submissive 1	2	Dominant	1
5	Submissive 2	2	Dominant	0.9993226
5	Dominant	3	Dominant	0.9999547
5	Submissive 1	3	Dominant	0.9999164
5	Submissive 2	3	Dominant	0.7299632
5	Dominant	4	Dominant	0.9950724
5	Submissive 1	4	Dominant	0.9965311
5	Submissive 2	4	Dominant	1
5	Submissive 1	5	Dominant	1
5	Submissive 2	5	Dominant	0.9931392
5	Submissive 1	1	Submissive 1	1
5	Submissive 2	1	Submissive 1	0.99839
5	Submissive 1	2	Submissive 1	0.9999976
5	Submissive 2	2	Submissive 1	0.8521544
5	Submissive 1	3	Submissive 1	0.946458
5	Submissive 2	3	Submissive 1	1

5	Submissive 1	4	Submissive 1	0.978406
5	Submissive 2	4	Submissive 1	1
5	Submissive 2	5	Submissive 1	0.9950724
5	Submissive 2	1	Submissive 2	0.9976096
5	Submissive 2	2	Submissive 2	1
5	Submissive 2	3	Submissive 2	0.946458
5	Submissive 2	4	Submissive 2	1

Journal of Experimental Biology • Supplementary information
